# The long-range interaction between two *GNAS* imprinting control regions delineates pseudohypoparathyroidism type 1B pathogenesis

**DOI:** 10.1172/JCI167953

**Published:** 2023-04-17

**Authors:** Yorihiro Iwasaki, Cagri Aksu, Monica Reyes, Birol Ay, Qing He, Murat Bastepe

**Affiliations:** 1 Endocrine Unit, Department of Medicine, Massachusetts General Hospital and Harvard Medical School, Boston, Massachusetts, USA.; 2 Tazuke Kofukai Medical Research Institute, Kitano Hospital, Osaka, Japan.; 3 The State Key Laboratory Breeding Base of Basic Science of Stomatology and Key Laboratory for Oral Biomedicine of the Ministry of Education, School and Hospital of Stomatology, Wuhan University, Wuhan, China.

**Keywords:** Endocrinology, Genetics, Embryonic stem cells, Epigenetics, Imprinting

## Abstract

Genetic defects of *GNAS*, the imprinted gene encoding the stimulatory G protein α-subunit, are responsible for multiple diseases. Abnormal *GNAS* imprinting causes pseudohypoparathyroidism type 1B (PHP1B), a prototype of mammalian end-organ hormone resistance. Hypomethylation at the maternally methylated *GNAS* A/B region is the only shared defect in patients with PHP1B. In autosomal dominant (AD) PHP1B kindreds, A/B hypomethylation is associated with maternal microdeletions at either the *GNAS* NESP55 differentially methylated region or the *STX16* gene located approximately 170 kb upstream. Functional evidence is meager regarding the causality of these microdeletions. Moreover, the mechanisms linking A/B methylation and the putative imprinting control regions (ICRs) NESP-ICR and STX16-ICR remain unknown. Here, we generated a human embryonic stem cell model of AD-PHP1B by introducing ICR deletions using CRISPR/Cas9. With this model, we showed that the NESP-ICR is required for methylation and transcriptional silencing of A/B on the maternal allele. We also found that the SXT16-ICR is a long-range enhancer of NESP55 transcription, which originates from the maternal NESP-ICR. Furthermore, we demonstrated that the STX16-ICR is an embryonic stage–specific enhancer enabled by the direct binding of pluripotency factors. Our findings uncover an essential *GNAS* imprinting control mechanism and advance the molecular understanding of PHP1B pathogenesis.

## Graphical abstract

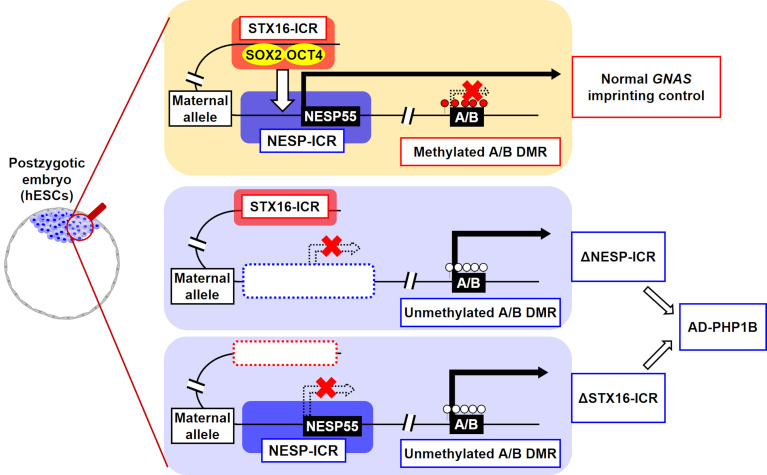

## Introduction

Genomic imprinting refers to epigenetic alterations that allow genes to be expressed monoallelically in a parent-of-origin–specific manner. The imprinted expression pattern primarily depends on cytosines at CpG dinucleotides that are differentially methylated between maternal and paternal alleles (1). The methylation of imprinted genes is established and maintained during gametogenesis and early embryonic development (2, 3). Aberrant methylation of a specific gene or a gene cluster can lead to loss or overexpression of the gene products depending on the affected parental allele. Such alterations are the basis of multiple developmental and/or endocrine diseases (1–3). However, mechanisms regulating genomic imprinting are poorly understood. Moreover, the molecular pathogenesis has been elucidated for few imprinting disorders (2).

**Figure 1:**
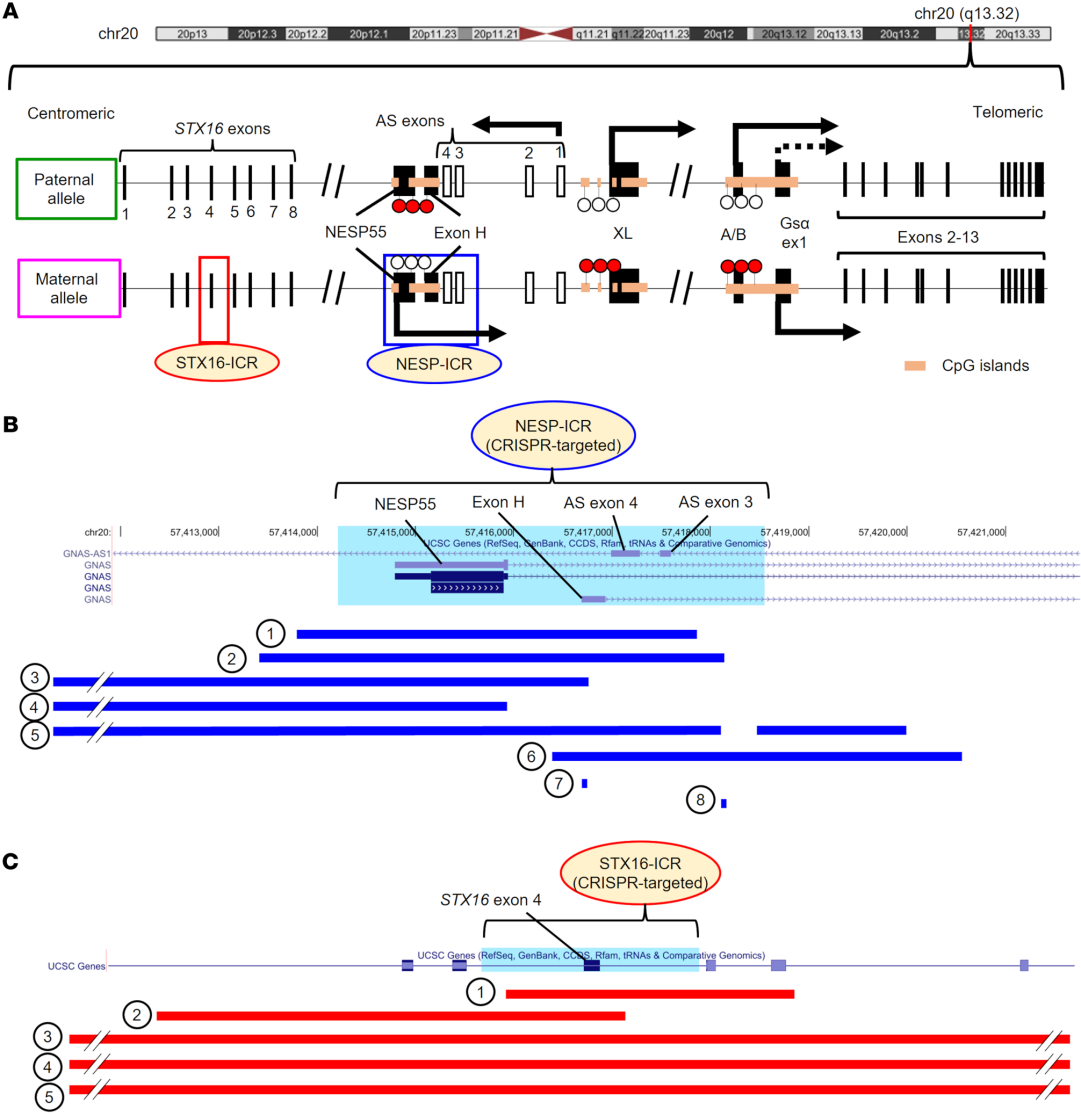
Putative *GNAS* ICRs and microdeletions identified in patients with AD-PHP1B. (**A**) Schematic locations of *STX16*, *GNAS*, and putative *GNAS* ICRs. Each box represents the targeted region to generate ICR-deleted hESC clones. White (unmethylated) and red (methylated) lollipops represent CpGs. Arrows show transcription from each exon, with a dotted arrow indicating silencing of Gsα expression in a tissue-specific manner. (**B** and **C**) Distribution of microdeletions in patients with AD-PHP1B with the NESP-ICR (**B**) or STX16-ICR (**C**) deletion. Each deletion is shown with a blue (**B**) or red (**C**) horizontal bar. Each ICR targeted by CRISPR/Cas9 is highlighted in light blue (GRCh37 chr20:57,414,216-57,418,552 and GRCh37 chr20:57,243,339-57,245,500 for the NESP-ICR and the STX16-ICR, respectively). The number on the left of each deletion corresponds to the number in Supplemental Tables 1 and 2, where the detailed information is described. For **A** and **C**, *STX16* exon numbers are based on NCBI RefSeq NM_003763.6.

*GNAS* is a complex imprinted locus encoding the α-subunit of the stimulatory G protein (Gsα), a ubiquitous signaling protein essential for the actions of numerous hormones, neurotransmitters, and autocrine and paracrine molecules (Figure 1A). Genetic defects affecting *GNAS* are responsible for several human diseases (4–9). Mutations causing constitutive Gsα activity are found in multiple benign and malignant tumors and cause McCune-Albright syndrome (7–9). Inactivating Gsα mutations lead to multihormone resistance, including end-organ resistance to the parathyroid hormone (PTH), termed pseudohypoparathyroidism (PHP) (4–7).

*GNAS* comprises at least 3 exons upstream of the Gsα-coding exons 1–13: NESP55, XL, and A/B, which are located within differentially methylated regions (DMRs) (Figure 1A). Each upstream exon has its own promoter and shows an imprinted expression pattern due to the presence of CpG islands that are differentially methylated (4, 6, 7). NESP55 has a paternally methylated promoter and shows maternal expression. XLαs (encoded by the exon XL) and A/B are paternally expressed due to maternal methylation of their promoters. Maternal methylation is also present at the promoter of an antisense transcript (AS) expressed paternally. While the Gsα promoter lacks methylation, Gsα expression is paternally silenced in a limited number of tissues, such as the proximal renal tubule, the thyroid gland, and the brown adipose tissue (10–12). Consistent with this complex imprinted profile of gene expression, most *GNAS*-related disease phenotypes are inherited in a parental origin-specific manner. For example, hormone resistance develops only if an inactivating mutation is located on the maternal *GNAS* allele (4–7). In addition, both maternal and paternal uniparental disomies involving the *GNAS* locus are disease causing (4–7, 13, 14). The mechanisms controlling the imprinting of *GNAS* are poorly defined.

PHP is characterized by hypocalcemia and hyperphosphatemia in the presence of elevated serum PTH, along with Albright’s hereditary osteodystrophy (AHO) in some cases (4–7, 15). The underlying cause is diminished Gsα activity due to either Gsα coding mutations or imprinting defects, corresponding to PHP1A and PHP1B subtypes, respectively (4–7, 16, 17). In PHP1B, the hormone resistance is largely confined to PTH, and AHO features are limited. Among *GNAS* imprinting abnormalities, hypomethylation at the A/B DMR is the only shared epigenetic defect in all reported patients with PHP1B (5, 6, 18). Thus, maternal A/B hypomethylation is the essential *GNAS* imprinting defect in PHP1B, and the regulatory mechanism of A/B methylation is the key to understanding its pathogenesis.

PHP1B can be sporadic or familial. Although the genetic basis of most sporadic PHP1B cases is unknown, except for rare patients with paternal uniparental disomy of chromosome 20, familial cases show an autosomal dominant maternal transmission (AD-PHP1B). Previous studies have identified chromosomal microdeletions in 2 different loci from AD-PHP1B kindreds. The first locus is a region within the *STX16* gene, located 170 kb centromeric of the *GNAS* locus (19). The second is the most centromeric portion of *GNAS*, including exon NESP55 (20). Deletions in either locus result in abnormal *GNAS* imprinting only when present on the maternal allele. Therefore, these 2 loci are putative imprinting control regions (ICRs) of *GNAS* that act specifically on the maternal allele (hereafter referred to as STX16-ICR and NESP-ICR, respectively). However, the mechanisms underlying their actions and the molecular relationship between these putative ICRs and PHP1B pathogenesis have hitherto remained unknown.

Studies using putative ICR-deleted murine models have yielded limited findings. Mice with a targeted deletion of the cognate STX16-ICR region did not recapitulate human *GNAS* imprinting defects, suggesting an interspecies difference in chromosomal positioning of putative *GNAS* ICRs between humans and mice (21). While deleting NESP-ICR in mice led to *GNAS* imprinting defects, it unexpectedly caused early postnatal lethality, which hampered further mechanistic studies (22). It thus remains unclear at what developmental stages and through which mechanisms STX16-ICR and NESP-ICR maintain A/B methylation. Therefore, to clarify the mechanisms regulating *GNAS* imprinting, a human cellular model that faithfully recapitulates *GNAS* imprinting defects in PHP1B is required. In this study, we generated human embryonic stem cell (hESC) models of AD-PHP1B and investigated the mechanistic basis of epigenetic defects in PHP1B. We revealed a critical long-range interaction between the 2 *GNAS* ICRs that controlling methylation at A/B in hESCs. Our findings provide functional evidence for the genetic and epigenetic mechanisms underlying PHP1B molecular pathogenesis and shed light on the regulation of genomic imprinting.

## Results

### Generation of AD-PHP1B model hESC clones with maternal NESP-ICR deletion.

To investigate the roles of putative *GNAS* ICRs, we reviewed the deletions reported in AD-PHP1B kindreds, which were located either in the region surrounding the *GNAS* NESP55 exon or the *STX16* locus (Figure 1, A–C, and Supplemental Tables 1 and 2; supplemental material available online with this article; https://doi.org/10.1172/JCI167953DS1). Based on the distribution of deletions, the putative NESP-ICR includes the NESP55, AS4, and AS3 exons and the intervening intronic sequences (Figure 1B and Supplemental Table 1). The putative STX16-ICR includes *STX16* exon 4 and the adjacent portion of intron 4 (exon/intron numbering according to NCBI RefSeq NM_003763.6) (Figure 1C and Supplemental Table 2). Maternal genomic imprints are critically regulated in the early embryo before implantation (2, 3). Thus, to clarify the roles of these putative *GNAS* ICRs at this developmental stage, we deleted each ICR in hESCs using CRISPR/Cas9 and isolated single-cell clones.

**Figure 2:**
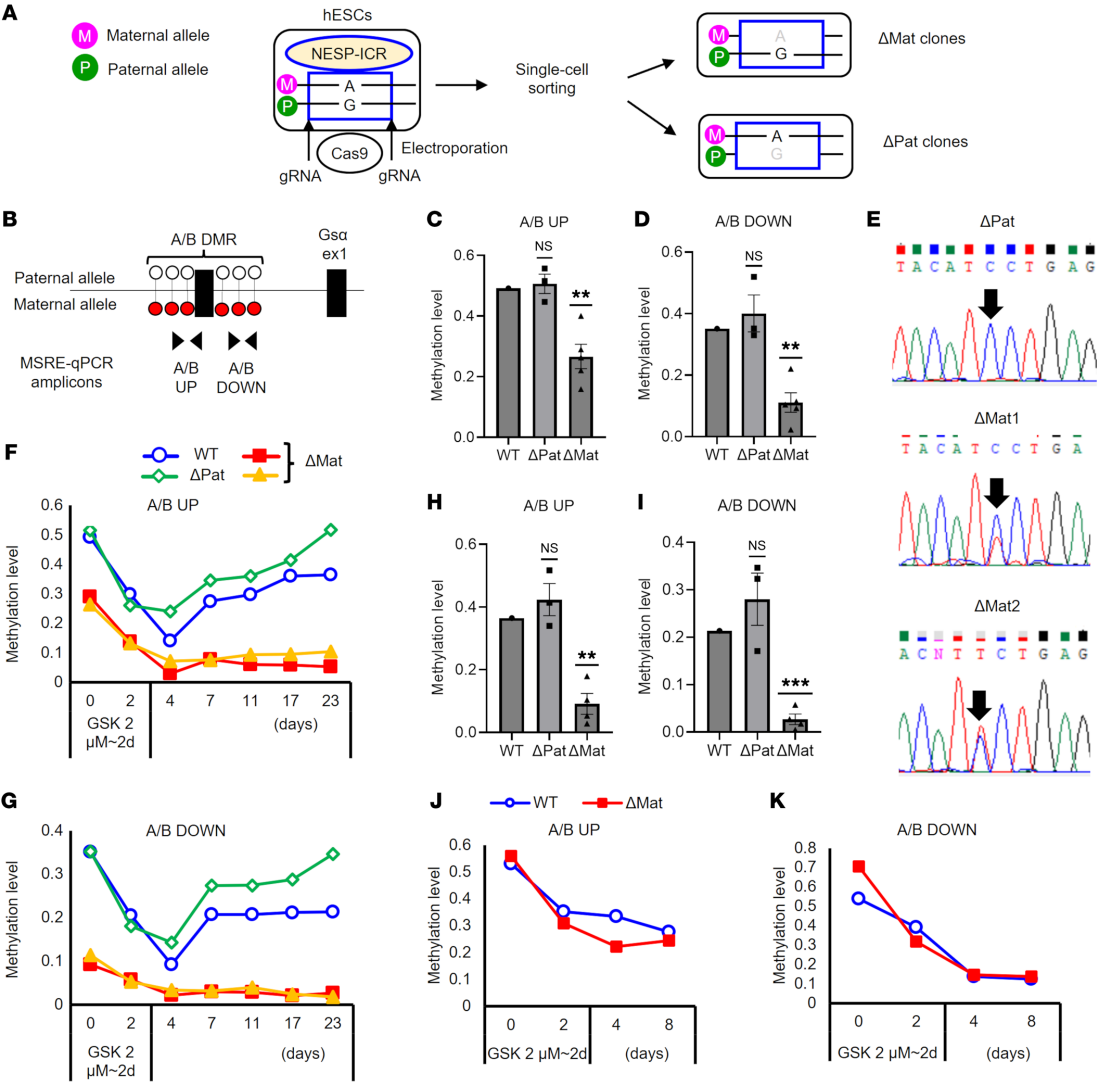
Characterization of NESP-ICR–deleted hESCs and HCT116 cells. (**A**) Experimental workflow for the generation of hESCs with NESP-ICR deletion. Following the introduction of Cas9 protein and gRNAs by electroporation, hESCs were single-cell sorted. Based on a heterozygous SNP (rs3787497, A/G), hESC clones were classified into maternally deleted (ΔMat) or paternally deleted (ΔPat) clones. (**B**) Schematics showing UP and DOWN MSRE-qPCR amplicons in the A/B DMR. White (unmethylated) and red (methylated) lollipops represent CpGs. (**C** and **D**) Baseline methylation levels at UP (**C**) and DOWN (**D**) amplicons were calculated by MSRE-qPCR in WT hESCs, three NESP-ICR ΔPat, and five ΔMat hESCs clones. (**E**) Sequencing of a *GNAS* exon 5 SNP (rs7121) in A/B transcripts. Three ΔPat and four ΔMat clones were analyzed, and representative results are shown. (**F**–**K**) Following the treatment with 2 μM GSK3484862 for 2 days, methylation levels were measured at the indicated time points by MSRE-qPCR. Time courses of the methylation levels at UP (**F**) and DOWN (**G**) amplicons in WT hESCs, one ΔPat, and two ΔMat clones. UP (**H**) and DOWN (**I**) amplicons at day 23 in WT hESCs, three ΔPat, and four ΔMat clones. Time courses of the methylation levels at UP (**J**) and DOWN (**K**) amplicons in WT and ΔMat HCT116 cells. For **C**, **D**, **H**, and **I**, each dot represents an independent hESC clone. WT versus ΔPat or ΔMat clones were compared using a 1-sample *t* test with Bonferroni correction for multiple comparisons. **P < 0.01 and ***P < 0.001.

First, we generated hESC clones with a heterozygous NESP-ICR deletion (Figure 2A). We designed guide RNAs (gRNAs) so that the deleted region (GRCh37 chr20:57,414,216-57,418,552) at least partially overlapped with all previously reported deletions within *GNAS* (Figure 1B). To determine the allelic origin of the deleted allele (i.e., paternal vs. maternal), which is critical for examining imprinting control effects, we used a heterozygous SNP (rs3787497; A/G) within the NESP-ICR (Figure 2A and Supplemental Figure 1A). This SNP was in a previously uncharacterized exon approximately 700 bp downstream of exon NESP55, which we tentatively named “exon H” according to the name of the presumably encoded isoform (NP_001296790.1). To determine the parent of origin of this exon H–containing transcript, we first examined each previously characterized *GNAS*-derived transcript using another heterozygous SNP (rs7121; C/T) in *GNAS* exon 5 (Supplemental Figure 1A). Sequence analysis of rs7121 in reverse transcription PCR (RT-PCR) products showed that, although the NESP55 transcript had only T, the A/B transcript had only C, indicating that the T allele was maternal (Supplemental Figure 1, B and C). Gsα expression was biallelic, and XLαs expression was also biallelic, albeit with a marked paternal bias (Supplemental Figure 1, D and E). The exon H–containing transcript showed only the T allele, and therefore, it was expressed exclusively from the maternal allele, at least in hESCs (Supplemental Figure 1F). Then, we further sequenced the SNP within exon H (rs3787497) in the exon H–containing transcript, which showed the A allele exclusively, indicating that the A allele must be maternal (Figure 2A and Supplemental Figure 1G). Based on this information, we determined the allelic origin of the deleted allele in NESP-ICR heterozygously deleted hESCs and obtained both maternally (NESP-ICRΔM) and paternally (NESP-ICRΔP) deleted clones (Figure 2A and Supplemental Figure 1H).

### The NESP-ICR is necessary for maternal A/B methylation and silencing.

To clarify the role of the NESP-ICR in A/B imprinting, we quantified A/B methylation levels by methylation-sensitive restriction enzyme quantitative PCR (MSRE-qPCR), as previously described (23). Consistent with differential methylation, WT hESCs showed 49.2% and 35.1% methylation at upstream (UP) and downstream (DOWN) amplicons in the A/B DMR, respectively (Figure 2, B–D). Notably, at baseline, NESP-ICRΔM clones, but not NESP-ICRΔP clones, showed significantly lower methylation levels at A/B UP and A/B DOWN amplicons, compared with WT hESCs (Figure 2, B–D). We also measured baseline A/B methylation levels using PCR amplicon sequencing of bisulfite-converted genomic DNA, which showed modest, albeit statistically significant, hypomethylation in a NESP-ICRΔM clone compared with WT (Supplemental Figure 2, A and B). Accordingly, the A/B transcript showed biallelic expression in NESP-ICRΔM clones, whereas it was exclusively paternally expressed in NESP-ICRΔP clones (Figure 2E). Gsα expression was not reduced and remained biallelic in NESP-ICRΔM clones (Supplemental Figure 3, A and B). In NESP-ICRΔM clones, the methylation level was not reduced at XL or AS DMRs, although the latter appeared modestly hypermethylated (Supplemental Figure 3, C and D). These results suggested that the NESP-ICR was required for methylation and transcriptional silencing of A/B on the maternal allele.

To further explore how the NESP-ICR affects A/B methylation, we examined our hESC clones under conditions mimicking the postzygotic embryonic period. CpG methylation at imprinted loci is established in a parent-of-origin–specific manner during gametogenesis (2, 3). After fertilization, the zygotic genome undergoes global demethylation in the preimplantation period (2, 3). However, methylation at imprinted loci shows resistance against this demethylation wave, thus allowing germline DMRs to be transmitted to differentiated cells (2, 3). Postzygotic demethylation on the maternal genome, on which A/B DMR is methylated, occurs passively because of a reduction in the activity of the maintenance methylase DNMT1 (2). To recapitulate this passive maternal demethylation process in vitro, we treated hESCs with a recently developed selective and reversible DNMT1 inhibitor, GSK-3484862 (24), and quantified A/B methylation levels. Unexpectedly, following a 2-day treatment with GSK-3484862, methylation levels at the A/B DMR were substantially decreased (14.1% and 9.2% methylation for UP and DOWN, respectively) in hESCs by day 4 (Supplemental Figure 4). After the removal of the inhibitor, methylation levels increased gradually (up to 36.5% and 21.4% methylation for UP and DOWN, respectively) (Supplemental Figure 4). In contrast, methylation levels at representative maternally methylated imprinted loci, *MCTS2*, *KCNQ1OT1*, and *PEG10*, showed resistance against DNMT1 inhibition–induced demethylation (Supplemental Figure 4). Consistent with these findings, recent methylome data on human and murine preimplantation embryos showed reduced methylation levels at the A/B (and its murine counterpart termed 1A) DMR compared with the levels of many other imprinted loci and those observed at later developmental stages (25, 26). Together, these findings suggested that *GNAS* A/B was an atypical imprinted locus whose methylation level is sensitive to lowered DNMT1 activity.

Using the above-mentioned strategy to mimic postzygotic methylation changes, we next compared demethylation and remethylation phases among NESP-ICRΔM, NESP-ICRΔP, and WT hESCs. The NESP-ICRΔM clones completely failed to remethylate the A/B DMR, unlike NESP-ICRΔP clones and WT hESCs, in which A/B DMR methylation markedly increased over approximately 3 weeks (Figure 2, F–I). We also confirmed the significant hypomethylation of the A/B DMR in a NESP-ICRΔM clone using PCR amplicon sequencing of bisulfite-converted genomic DNA (Supplemental Figure 2, C and D). These results demonstrated that the NESP-ICR on the maternal allele was necessary during remethylation at the A/B DMR in hESCs. By contrast, in a somatic colon cancer cell line (HCT116 cells), maternal deletion of the NESP-ICR did not lead to clear A/B methylation changes, suggesting that the NESP-ICR was no longer required for A/B methylation in differentiated cells (Figure 2, J and K, and Supplemental Figure 1I).

### The STX16-ICR is necessary for A/B methylation and NESP55 transcription.

**Figure 3:**
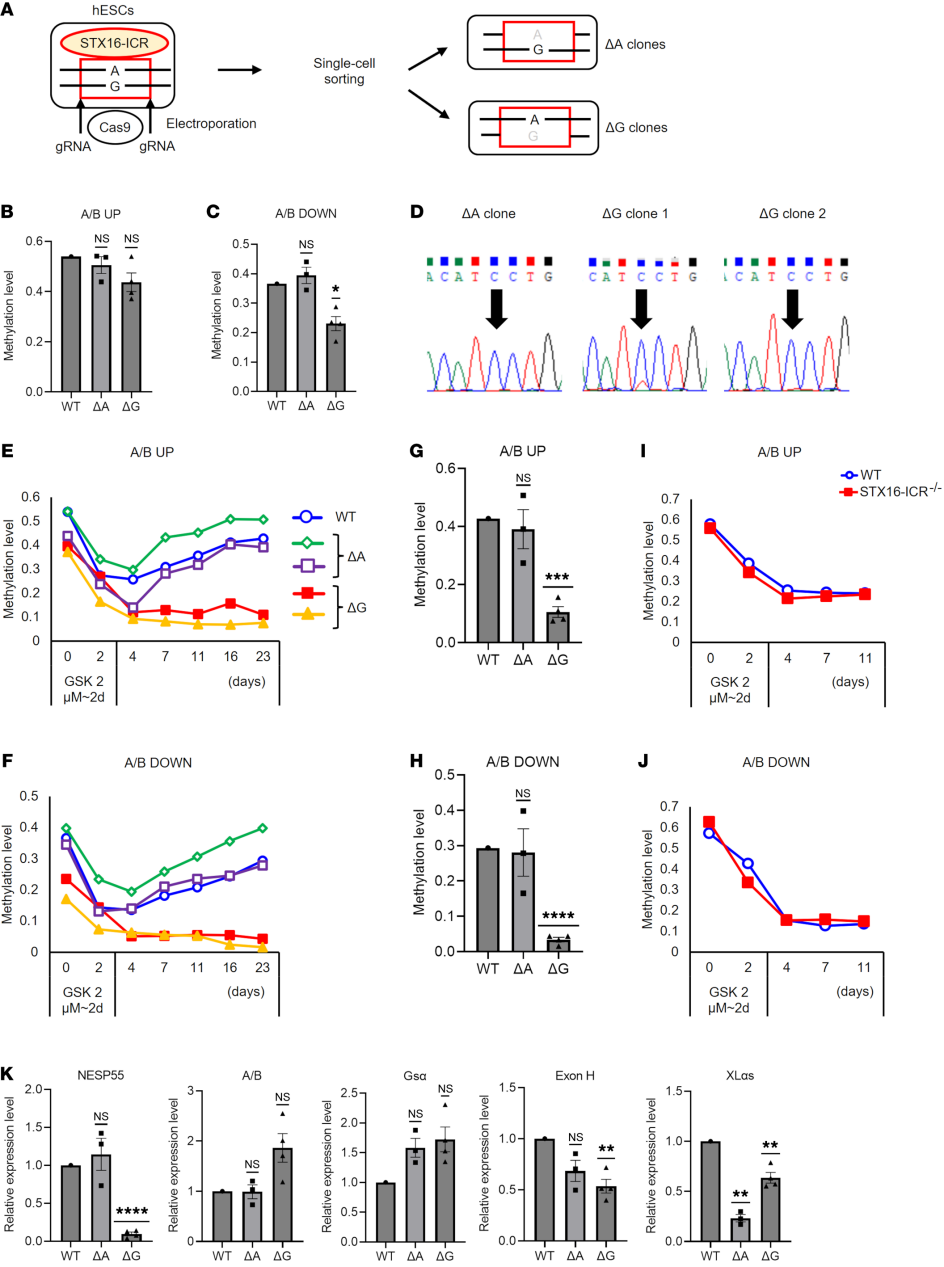
Generation and characterization of STX16-ICR–deleted hESCs and HCT116 cells. (**A**) Experimental workflow for generating STX16-ICR–deleted hESCs. An SNP (rs2296524, A/G) was used to distinguish A allele–deleted (ΔA) or G allele–deleted (ΔG) clones. (**B** and **C**) Baseline A/B methylation levels at UP (**B**) and DOWN (**C**) amplicons were calculated by MSRE-qPCR in WT hESCs, 3 STX16-ICR ΔA hESC clones, and 4 ΔG hESC clones. (**D**) Sequencing of a *GNAS* exon 5 SNP (rs7121) in A/B transcripts. Three ΔA and 4 ΔG hESC clones were analyzed: 1 representative ΔA clone and 2 representative ΔG clones are shown. (**E**–**J**) Following the treatment with 2 μM GSK3484862 for 2 days, A/B methylation levels were calculated at the indicated time points by MSRE-qPCR. Time courses of the methylation levels at UP (**E**) and DOWN (**F**) amplicons in WT hESCs, 2 ΔA hESC clones, and 2 ΔG hESC clones. UP (**G**) and DOWN (**H**) amplicons on day 23 in WT hESCs, 3 ΔA hESC clones, and 4 ΔG hESC clones. Time courses of the methylation levels at UP (**I**) and DOWN (**J**) amplicons in WT and STX16-ICR^–/–^ HCT116 cells. (**K**) Expression levels of *GNAS* transcripts in WT, ΔA, and ΔG hESCs, quantified by qRT-PCR and normalized to β-actin. For **B**, **C**, **G**, **H**, and **K**, each dot represents an independent hESC clone. WT hESCs versus ΔA or ΔG hESC clones were compared using a 1-sample *t* test with Bonferroni correction for multiple comparisons. *P < 0.05, **P < 0.01, ***P < 0.001, and ****P < 0.0001.

To elucidate the role of the STX16-ICR, we then generated hESC clones with STX16-ICR (GRCh37 chr20:57,243,339–57,245,500) deletions. Using a heterozygous A/G SNP within the STX16-ICR (rs2296524) for allelic determination, we successfully generated both A allele–deleted (STX16-ICRΔA) and G allele–deleted (STX16-ICRΔG) hESC clones (Figure 3A). We could not directly determine the deleted allele’s parental origin because the *STX16* gene was nonimprinted in hESCs (Supplemental Figure 5, A–C), consistent with a previous study using human lymphoblastoid cells (27). At baseline, STX16-ICRΔG clones showed significantly lower methylation levels compared with WT hESCs at the A/B DOWN region, although the reduction of methylation at the A/B UP region was not statistically significant (Figure 3, B and C). A/B methylation levels in STX16-ICRΔA clones were not significantly different from the levels in WT hESCs. The A/B transcript showed biallelic, albeit highly paternally skewed, expression in 1 of 4 STX16-ICRΔG clones (ΔG clone 1), whereas it was exclusively paternally expressed in STX16-ICRΔA clones (Figure 3D). Gsα expression remained biallelic in STX16-ICRΔG clones (Supplemental Figure 6A). In STX16-ICRΔG clones, the methylation level was not reduced at XL or AS DMRs, although the latter appeared modestly hypermethylated (Supplemental Figure 6, B and C). Following DNMT1 inhibition, STX16-ICRΔG hESC clones failed to regain methylation at the A/B DMR, as opposed to STX16-ICRΔA clones or WT hESCs, which displayed marked remethylation (Figure 3, E–H). By contrast, HCT116 cells with STX16-ICR homozygous deletion (STX16-ICR^–/–^) did not show defects in A/B methylation (Figure 3, I and J). The clear difference in A/B methylation between STX16-ICRΔG and ΔA clones strongly suggested that the G allele was putatively maternal and that STX16-ICR affects A/B methylation on this allele.

The defective A/B methylation observed in STX16-ICRΔG clones was reminiscent of the phenotype of NESP-ICRΔM clones. Since mouse studies implicated Nesp55 transcription in the regulation of *Gnas* imprinting (22, 28), we measured NESP55 transcript expression in STX16-ICRΔG and STX16-ICRΔA clones using qRT-PCR. Strikingly, NESP55 transcript levels in STX16-ICRΔG clones were only approximately 10% of the levels in WT hESCs, suggesting that the STX16-ICR may regulate NESP55 transcription in hESCs (Figure 3K). In contrast, STX16-ICRΔG clones showed increased AB expression, although the difference did not reach statistical significance (*P* = 0.1124 after Bonferroni correction). Gsα transcript levels were not significantly altered in STX16-ICRΔG clones (Figure 3K), an expected finding given the biallelic expression of Gsα in both WT hESCs (Supplemental Figure 1D) and STX16-ICRΔG clones (Supplemental Figure 6A). Exon H–containing transcript levels were also modestly decreased in STX16-ICRΔG clones. Interestingly, XLαs transcript levels were decreased in STX16-ICRΔA clones and, to a lesser extent, in STX16-ICRΔG clones (Figure 3K).

### The STX16-ICR is a long-range enhancer of the NESP55 promoter in hESCs.

**Figure 4:**
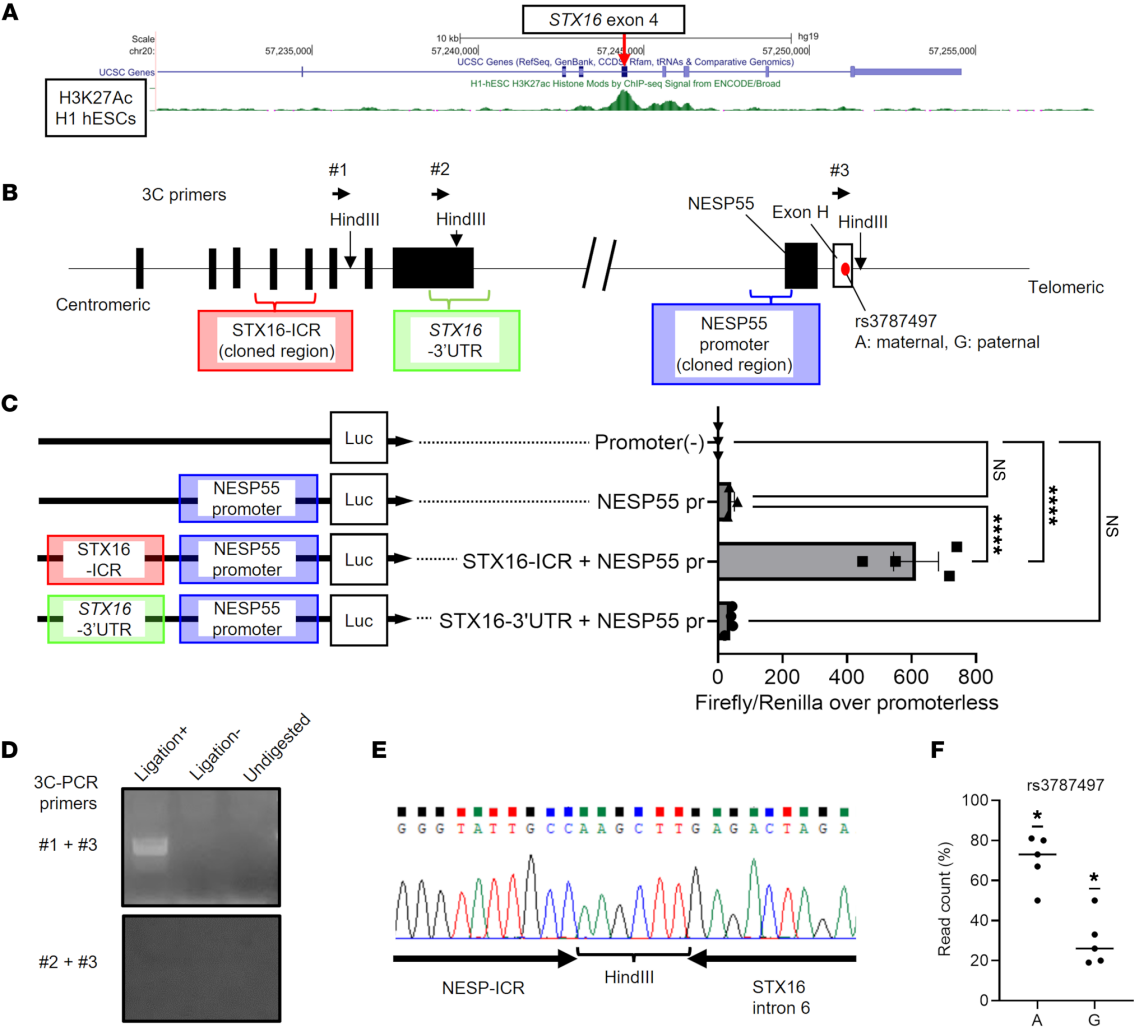
Long-range interaction between the STX16-ICR and the NESP-ICR. (**A**) Genome Browser track showing H3K27Ac ChIP-Seq signals within the *STX16* locus in H1 hESCs. Exon numbering is based on NCBI RefSeq NM_003763.6. (**B**) Schematic representation of cloned regions for luciferase assays and primer locations used in 3C assays. (**C**) Luciferase assay in WT hESCs using a negative control [promoter(–)] or NESP55 promoter (NESP55pr, blue box), NESP55pr plus STX16-ICR (red box,) or *STX16*–3′-UTR (light green box) constructs (left). Graph shows a representative result of 5 independent experiments. Intergroup comparisons were performed by 1-way ANOVA with Tukey’s post hoc test. ****P < 0.0001. (**D**–**F**) 3C PCR assay in hESCs. Proximity-ligated fragments were amplified using primers #1–#3, as depicted in **B**. (**D**) PCR results from 3C samples (Ligation+) and negative control templates, i.e., no ligation (Ligation–) and no digestion (Undigested) using primers #1 and #3 (upper gel), and primers #2 and #3 (lower gel, a negative control locus). Representative images from 5 independent experiments are shown. (**E**) Sequencing of the 3C-PCR product using primers #1 and #3 showing the ligation junction. (**F**) Percentages of A (maternal) and G (paternal) NGS reads at rs3787497 in 3C PCR products. Each dot represents an independent experiment. The interaction frequency was statistically compared with 50% (biallelic) by a 1-sample *t* test with Bonferroni correction. *P < 0.05.

Since the STX16-ICR is located in a region approximately 170 kb centromeric of the NESP55 exon, we hypothesized that the STX16-ICR might be an enhancer of NESP55 transcription. We referred to the Encyclopedia of DNA Elements (ENCODE) database, which showed the enrichment of lysine 27–acetylated histone H3 (H3K27Ac) mark within the STX16-ICR in hESCs (Figure 4A). This finding supported our hypothesis, as this chromatin mark is associated with enhancer elements. On the other hand, we did not observe H3K27Ac enrichment in the corresponding *Stx16* region in murine ES cells (Supplemental Figure 7). To test the enhancer role of the human STX16-ICR, we cloned the putative NESP55 promoter and the STX16-ICR region around the H3K27Ac mark from human genomic DNA and performed luciferase reporter assays using WT hESCs (Figure 4, B and C). The NESP55 promoter alone showed increased luciferase activity compared with the promoterless construct (42-fold), although statistical significance could not be reached with the ANOVA and multigroup comparisons with Tukey’s post hoc test. By contrast, the construct containing the STX16-ICR showed a further significant enhancement of NESP55 promoter–driven luciferase activity (610-fold vs. promoterless) (Figure 4C). The construct with a control region around the *STX16* 3′-UTR did not enhance NESP55 promoter activity (Figure 4C).

In addition, chromosomal conformation capture (3C) assays in WT hESCs allowed amplification of specific PCR products, using primers #1 (*STX16* intron 6) and #3 (NESP-ICR), demonstrating that the STX16-ICR and NESP-ICR are in close proximity (Figure 4, B, D, and E). In contrast, the interaction was not captured using primers #2 (*STX16* 3′-UTR) or #3 (NESP-ICR) (Figure 4D). To identify the parental origin of the captured allele, we used the SNP in exon H, located within the NESP-ICR (rs3787497, A/G), for which A is maternal (Supplemental Figure 1, A, F, and G). Next-generation sequencing (NGS) analysis of the 3C PCR products revealed that the sequencing reads were significantly skewed from equal distribution to the A allele (Figure 4F), demonstrating a bias in the interaction between the STX16-ICR and the NESP-ICR toward the maternal allele, from which NESP55 is expressed. These results strongly supported our hypothesis that the STX16-ICR operated as a long-range enhancer for the NESP55 promoter in hESCs.

### Pluripotency-associated factors are necessary for the enhancer function of the STX16-ICR.

**Figure 5:**
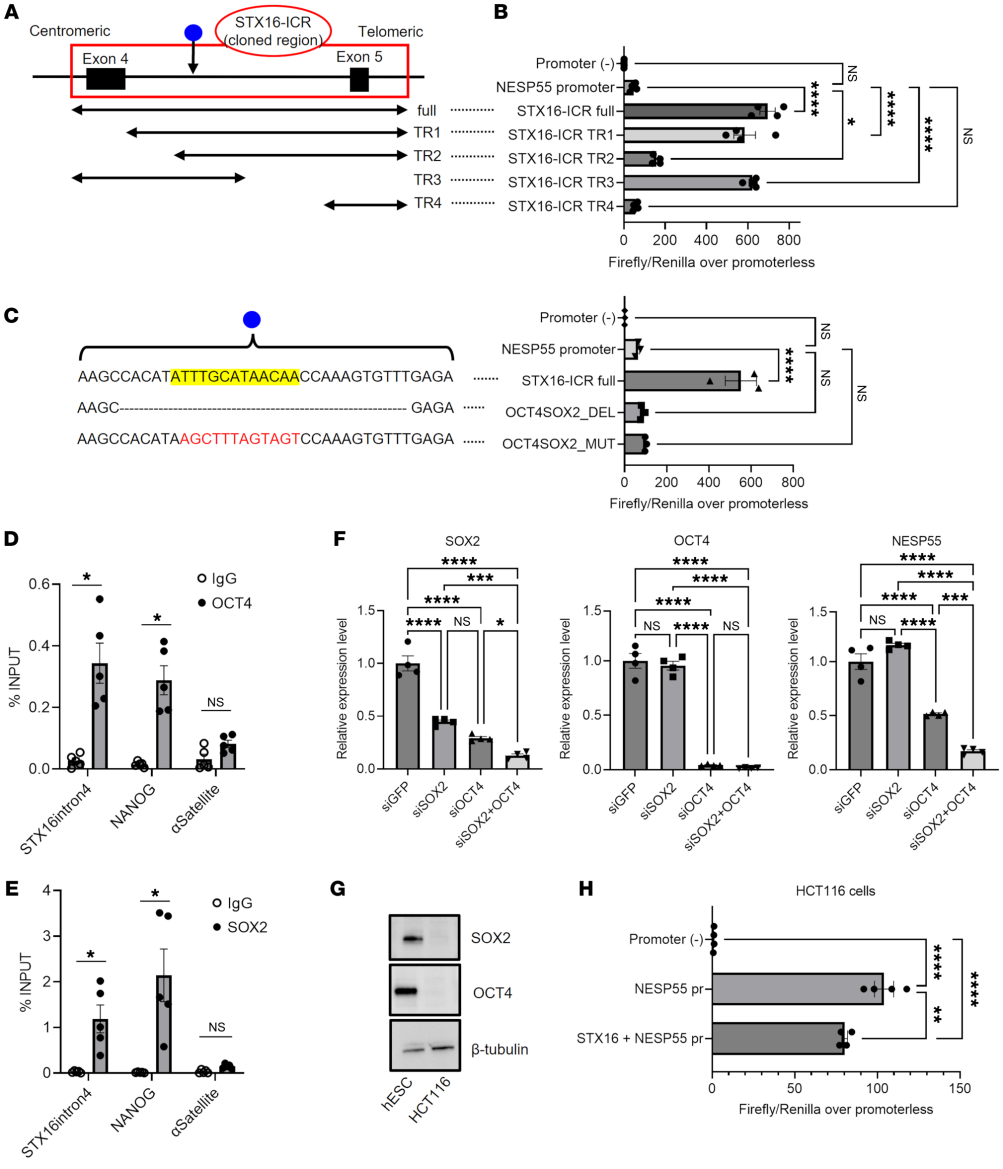
Pluripotency factors enable the STX16-ICR enhancer. (**A**–**C**) NESP55 promoter luciferase assay in WT hESCs. (**A**) Schematic representation of full-length (full) and truncated (TR1–4) STX16-ICR constructs. Blue circle indicates the putative OCT4/SOX2 motif location. (**B**) Results using the full-length and truncated constructs (TR1–4) of the STX16-ICR/NESP55 promoter luciferase vector. (**C**) Results using the full-length STX16-ICR and constructs in which an OCT4/SOX2 motif (yellow-highlighted) in *STX16* intron 4 (STX16-ICR full) was deleted (DEL) or mutated (MUT). (**D** and **E**) CUT&RUN qPCR analysis of OCT4 (**D**) and SOX2 (**E**) in WT hESCs. Primers on the OCT4/SOX2 site in *STX16* intron 4 (STX16intron4), NANOG promoter (positive control), and αSatellite (negative control) were used. Each dot represents 1 of 5 independent experiments. (**F**) Knockdown of OCT4 and/or SOX2 in WT hESCs. After transfection with siGFP (negative control), siOCT4, and/or siSOX2, the transcript levels of SOX2, OCT4, and NESP55 were quantified by qRT-PCR. (**G**) Western blotting of OCT4 and SOX2 in WT hESCs and HCT116 cells. β-Tubulin was used as a loading control. (**H**) Luciferase assay in HCT116 cells using negative control [promoter(–)], NESP55 promoter (NESP55 pr), and NESP55 pr plus STX16-ICR luciferase vectors. For **B**, **C**, **F** and **H**, representative results of 3 independent experiments are shown. Intergroup comparisons were performed by 1-way ANOVA with Tukey’s post hoc test. *P < 0.05,**P < 0.01, ***P < 0.001, and ****P < 0.0001.

To delineate the critical region for the enhancer function of the STX16-ICR, we generated serially truncated luciferase constructs (TR1–4) (Figure 5A). While the truncated construct missing most of *STX16* intron 4 (TR4) lost its enhancer effect, TR1 (missing *STX16* exon 4) and TR3 (missing the telomeric half of *STX16* intron 4) showed intact enhancer activity on the NESP55 promoter (Figure 5B). Construct TR2, which lacked exon 4 and the adjacent 200 bp of intron 4, still showed a significant, albeit blunted, enhancer effect (Figure 5B). These findings suggested that a portion of *STX16* intron 4, where the TR1 and TR3 overlap (GRCh37 chr20:57,244,524-57,245,119), was critical for the enhancer effect of the STX16-ICR. In addition, although a necessary sequence for the full enhancer effect was missing in TR2, the portion of TR2 overlapping with TR3 appeared to contain critical enhancer elements.

To identify the *trans*-acting factors enabling the STX16-ICR’s enhancer activity, we first referred to publicly available ChIP-Seq data obtained from H1 hESCs, which showed a signal peak in *STX16* intron 4, including signals for OCT4 and SOX2, well-characterized pluripotency-associated transcription factors (Supplemental Figure 8, A and B). In the region where TR2 and TR3 overlap within intron 4, we then identified a sequence highly homologous to the reported OCT4-SOX2 composite binding motif (GRCh37 chr20:57,244,895-57,244,907) (Figure 5A) (29). Deleting or mutating the OCT4-SOX2 motif in the full-length STX16-ICR luciferase construct resulted in an almost complete loss of STX16-ICR enhancer activity in hESCs (Figure 5C). Moreover, CUT&RUN qPCR assays in WT hESCs confirmed the recruitment of OCT4 and SOX2 to this region (Figure 5, D and E). Furthermore, siRNA-mediated knockdown of OCT4 alone or both OCT4 and SOX2 significantly reduced NESP55 transcript levels in WT hESCs (Figure 5F). In HCT116 cells, which lack OCT4 and SOX2 protein expression (Figure 5G), the STX16-ICR did not show any enhancer activity on the NESP55 promoter (Figure 5H). Consistent with this finding, the ENCODE database showed the presence of H3K27Ac in the STX16-ICR only in hESCs, not in somatic cells (Supplemental Figure 8C). These results not only revealed OCT4 and SOX2 as critical factors for the STX16-ICR but also explained the mechanistic basis of the pluripotent cell–specific enhancer activity of this region.

## Discussion

**Figure 6:**
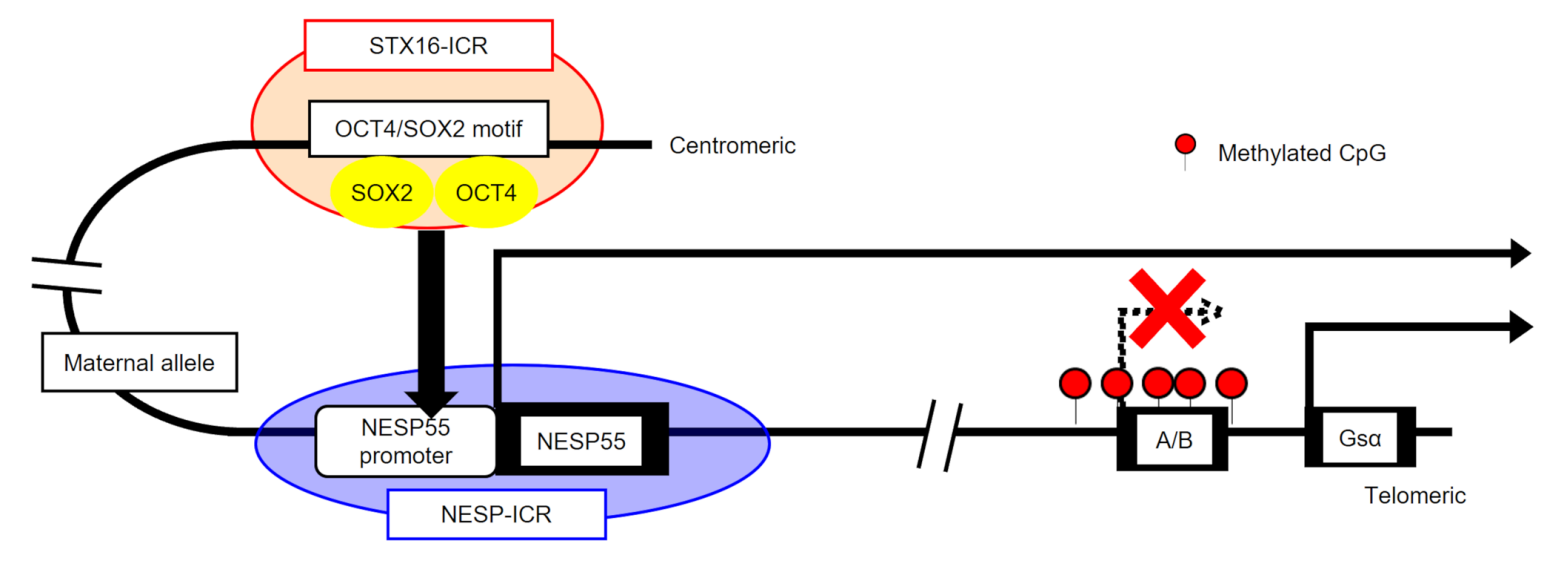
Schematic representation. The STX16-ICR is an early embryonic stage–specific long-range enhancer of maternal NESP55 transcription, which is required for A/B DMR methylation and A/B transcriptional silencing. In patients with AD-PHP1B, maternal deletion of either the STX16-ICR or the NESP-ICR disrupts the interaction of these control regions and thereby leads to hypomethylation at the A/B DMR and transcriptional derepression of A/B.

Since the definition of PHP as the prototypical hormone resistance disease in 1942 (15), it took several decades until impaired Gsα activity was found in some patients with PHP (16). Although Gsα coding mutations were later reported in patients with PHP1A (17), the etiology of PHP1B remained largely enigmatic until it was shown to be imprinted and associated with *GNAS* methylation abnormalities (30, 31). We identified deletions affecting *STX16* or NESP55 in AD-PHP1B kindreds in the 2000s (19, 20). Although all reported AD-PHP1B deletions reside in either of the 2 regions (5, 6, 18), the mechanistic explanation linking them to PHP1B pathogenesis has remained unclear. In this study, we used hESC models to investigate the molecular basis of the imprinting defects in AD-PHP1B, revealing *STX16* and NESP55 regions as essential ICRs for *GNAS* (Figure 6). Our study can be summarized into 3 main findings: (a) the essential role of the NESP-ICR in A/B methylation in the postzygotic period, (b) the long-range enhancer effect of the STX16-ICR on NESP55 transcription, and (c) the pluripotent cell–specific nature of the enhancer.

The only shared epigenetic defect in reported PHP1B cases is hypomethylation at the A/B DMR, highlighting the critical role of this DMR in the pathogenesis (5, 6, 18). Our results clearly show that the maternal NESP-ICR was required for A/B DMR methylation in hESCs, thus supporting the causal role of NESP-ICR deletions in AD-PHP1B. These deletions disrupt the NESP55 transcript, and it has been shown in mice that Nesp55 transcription is essential for maternal *Gnas* imprints (28). Recent studies of unrelated PHP1B patients also described genetic abnormalities such as retrotransposon insertion, inversion, or duplication in *GNAS* that potentially disrupted NESP55 transcription upstream of the A/B DMR (32–35). One of the studies demonstrated diminished NESP55 transcript levels in patient-derived induced pluripotent cells (33), consistent with the role of NESP55 transcription in A/B methylation. When and how does maternal NESP55 transcription affect the A/B DMR? In general, methylation at imprinted DMRs is introduced during gametogenesis (2, 3). According to a previous study in mice, methylation at the 1A (the murine counterpart of A/B) DMR is first introduced during oogenesis (36), similar to typical germline DMRs of maternally imprinted loci (26). Moreover, prematurely truncating Nesp55 transcription disrupts methylation at the 1A DMR in mouse oocytes (28). These findings collectively suggest that, at least in mice, NESP55 transcription is necessary for the establishment of 1A methylation during gametogenesis.

However, a striking characteristic of the A/B DMR is its reduced methylation levels during the postzygotic period. After fertilization, while passive genome-wide demethylation occurs on the maternal allele as a result of diminished DNMT1 activity, DMRs of imprinted genes generally resist this demethylation wave (2, 3). Nonetheless, human and murine methylome data indicate decreased A/B methylation in early postzygotic embryos (25, 26). Since the methylation level at the A/B DMR tends to be higher in somatic cells than pluripotent stem cells (25, 37), it is likely that the A/B DMR regains methylation in the postimplantation embryo. Our results using transient DNMT1 inhibition showed a similar pattern of demethylation and remethylation at the A/B DMR, which differed from the pattern at other representative maternally imprinted loci. Therefore, A/B imprinting should be considered atypical because it changes dynamically during an early postzygotic period.

Our NESP-ICRΔM hESC clones, in which NESP55 transcription was abolished, showed baseline hypomethylation and a complete lack of remethylation at the A/B DMR. Conversely, our previous study of an AD-PHP1B kindred revealed an association between derepressed paternal NESP55 transcription and partial gain of methylation on the paternal A/B region (38). In addition, a mouse study showed that ectopic paternal Nesp55 transcription results in gain of methylation at the 1A DMR (39). Since the paternal A/B DMR should be unmethylated in the zygote, these findings support the conclusion that NESP55 transcription can introduce postzygotic methylation at the A/B DMR in *cis*. Thus, the postzygotic embryonic stage, especially around the implantation period, is an essential regulatory phase of *GNAS* imprinting where NESP55 transcription is indispensable.

The function of another ICR, the STX16-ICR, has been completely unknown since the murine model of *STX16* deletion did not recapitulate the *GNAS* imprinting defects observed in AD-PHP1B (21). In the current study, we provide critical evidence that the STX16-ICR functions as a long-range enhancer for the NESP55 promoter, which is included in the NESP-ICR. Enhancer-mediated imprinting control is reported at another imprinted locus, the *IGF2/H19* locus, where an enhancer differentially acts on each parental allele: it activates *IGF2* and *H19* transcription on the paternal and maternal alleles, respectively (2). Similarly, our SNP analysis of 3C PCR products showed maternal predominance in the interaction between the STX16-ICR and the NESP-ICR, which is consistent with the NESP55 promoter being active on the maternal allele. The mechanistic basis of maternal predominance remains to be elucidated. Data from a publicly available ChIP-Seq database showed that CCCTC-binding factor (CTCF) recruitment to the NESP55 promoter could mediate allele-specific interaction between ICRs, as demonstrated for other loci (40), since its binding is blocked by CpG methylation (41). We further identified a small region of *STX16* intron 4 indispensable for embryonic stage–specific enhancer function. This region comprises a binding site for OCT4 and SOX2, which are pluripotency-associated transcription factors expressed abundantly during the postzygotic period. OCT4 and SOX2 can form a complex on enhancers in hESCs (29). Since the STX16-ICR did not show an enhancer effect in a somatic cell line, the STX16-ICR enhancer is likely to be active specifically during the early postzygotic period.

Our results showed that the STX16-ICR enhanced NESP55 transcription from the NESP-ICR in a postzygotic stage–specific manner, which controls methylation at the A/B DMR in this critical period of *GNAS* imprinting. Perturbation of this regulatory mechanism, either by genetic ICR deletions or by as-yet-undefined events may be the underlying cause of certain PHP1B cases. Patients with sporadic PHP1B often have incomplete *GNAS* methylation abnormalities (42–44). These abnormalities must reflect mosaicism, and studies showed that they are likely to be acquired during early postzygotic stages (42–44). Therefore, the mechanisms underlying the incomplete methylation alterations in sporadic PHPIB may include aberrant postzygotic regulation of *GNAS* imprinting in a subset of early embryonic cells.

At baseline, maternal STX16-ICR deletion was not as severe as the NESP-ICR deletion in terms of its effect on A/B methylation, although both deletions identically inhibited, almost completely, the regain of methylation. This difference at baseline suggests several possibilities. First, the suppression of NESP55 transcription upon maternal STX16-ICR deletion is not as robust as the maternal NESP-ICR deletion, which removes the NESP55 promoter and abrogates NESP55 transcription entirely. Although this might reflect the presence of additional regulatory mechanisms of NESP55 transcription (e.g., additional enhancers), it appears possible that the residual NESP55 transcription prevented a major loss of A/B methylation at baseline. Second, the STX16-ICR may play a more important role during the regain of A/B methylation than in its maintenance. Remarkably, our findings following DNMT1 inhibition suggest that this mechanism, i.e., STX16-ICR–regulated NESP55 transcription, critically orchestrates the remethylation of A/B in the blastocyst. Third, the STX16-ICR could also be important in developmental stages before the blastocyst, and therefore, its deletion in hESCs might have a limited effect regarding A/B methylation maintenance. On the other hand, our results also suggest that the STX16-ICR might regulate transcription from additional *GNAS* exons, especially XL and exon H. Given the decreased XLαs transcript levels in STX16-ICRΔA and, to a lesser extent, STX16-ICRΔG clones, the STX16-ICR might enhance XLαs transcription on both parental alleles. XLαs is paternally transcribed in most cells. Note that maternal XLαs transcription was not completely silenced in hESCs (see Supplemental Figure 1E), which is consistent with previous studies showing maternal allele contribution to XLαs in bone marrow stromal cells (45, 46).

Our findings on the role of the STX16-ICR also highlight the interspecies differences in imprinting control loci between humans and mice. While the structural organizations of murine *Gnas* and *Stx16* loci are similar to their human counterparts, the murine *Stx16* intron 4 sequence does not show homology to the human *STX16* intron 4. In addition, there was no enrichment of H3K27Ac in the mouse *Stx16* intron 4 region. These observations are consistent with previous findings in mice with deletion of *Stx16* exons 2–4, which lacked *Gnas* imprinting abnormalities or evidence of hormone resistance (21). The evolution of enhancers is reported to be much more rapid than that of promoters (47). Accordingly, the role of the NESP-ICR, which contains the evolutionarily conserved NESP55 promoter, was recapitulated in mice (22), whereas the role of the STX16-ICR was not (21). Several lines of evidence also suggest that imprinting control mechanisms in primates differ from those in rodents (48–50). Therefore, hESCs are an optimal tool for exploring the imprinting control mechanisms in humans. Our approach may apply to the study of other imprinting diseases.

Based on our results from the truncated reporter constructs, some unidentified factors, on top of OCT4 and SOX2, may also be required for the complete enhancer function of STX16-ICR. The truncated luciferase construct number 2 (TR2), which contains the OCT4/SOX2 site but lacks a 200 bp portion of *STX16* intron 4 that is included in TR1, showed substantially weaker enhancer activity than the latter, indicating that additional factors recruited to this region are also required. In addition, the OCT4/SOX2 site in *STX16* intron 4 is just outside of one of the previously identified *STX16* microdeletions (27). Since the telomeric breakpoint of this deletion is only 42 bp centromeric to the OCT4/SOX2 site, this deletion might still disturb the recruitment of OCT4 and SOX2. Alternatively, this deletion could disrupt the recruitment of the additional factors necessary for the enhancer function of the STX16-ICR. To our knowledge, no mutations or small insertions/deletions limited to the OCT4/SOX2 site in *STX16* intron 4 have yet been reported in patients with AD-PHP1B. A commonly used diagnostic approach using a methylation-sensitive, multiplex ligation–dependent probe amplification (MS-MLPA) assay is useful to quantitate copy numbers of *STX16* and *GNAS* exons and the methylation levels of *GNAS* DMRs (5). However, because of the probe design, it cannot detect such mutations or small deletions. Given our results, sequencing analysis of *STX16* intron 4 should be considered in patients with AD-PHP1B with isolated A/B loss of methylation, especially when MS-MLPA rules out deletions in this region.

Regarding the pathogenesis of PHP1B, several questions remain unanswered. First, although most patients with AD-PHP1B carrying NESP-ICR deletions have hypomethylation at the DMRs containing the promoters of AS and XL, we did not detect this alteration in our hESC clones with maternal NESP-ICR ablation. Thus, the methylation of those DMRs may be subject to different spatial and temporal constraints, such as germline mechanisms. In addition, maternal premature truncation of Nesp55 transcription in mice led to offspring with A/B hypomethylation, but abnormalities at other *Gnas* DMRs were variable (28), suggesting that some stochastic events or environmental factors, at least in mice, may add to the final methylation landscape. Second, Gsα silencing on the paternal allele takes place in a tissue-specific manner only in limited differentiated tissues (10–12). Accordingly, Gsα showed biallelic expression in hESCs (see Supplemental Figure 1D), and its levels were not affected by the individual ICR deletions and resultant alterations in A/B. Hence, an as-yet-unknown somatic tissue-specific mechanism is likely required for Gsα silencing, which is the basis for hormone resistance. Further studies using appropriate somatic cells are required to delineate the underlying tissue-specific mechanism.

No curative therapy is currently available for imprinting diseases, including PHP1B. The essential pathophysiological findings in our study may provide the framework for future therapeutic approaches to treating epigenetic defects in patients with PHP1B. Our results demonstrate that imprinting defects in AD-PHP1B can arise or evolve during the postzygotic period, in which the active long-range interaction between the STX16-ICR and the NESP-ICR is required for A/B DMR methylation. Therefore, an attempt to correct A/B DMR hypomethylation in this critical window would be a promising therapeutic approach for patients with AD-PHP1B. Furthermore, knowledge about the different roles of the STX16-ICR and the NESP-ICR provides the rationale for varying therapeutic targets in each AD-PHP1B case. Restoring NESP55 transcription, especially in patients with STX16-ICR deletion, may potentially reverse hypomethylation at the A/B DMR. We believe our AD-PHP1B model hESCs will be a powerful tool in the search for such therapeutic interventions.

## Methods

### Cell culture and genome editing.

Among hESC lines, we used HUES62 (NIH registration no. 0065) cells, since they showed heterozygosity in a SNP in *GNAS* exon 5 (rs7121, C/T), which enabled our allelic determination of *GNAS* transcripts. HUES62 cells were obtained from Harvard Stem Cell Research Institute and maintained in mTeSR1 Plus (STEMCELL Technologies) on 6-well culture plates precoated with Cultrex Cultrex Stem Cell Qualified Reduced Growth Factor Basement Membrane Extract (R&D Systems). Putative *GNAS* ICRs, ENCODE ChIP-Seq, and ENCODE histone data were visualized using the UCSC Genome Browser (http://genome.ucsc.edu). Genome editing of hESCs was performed according to a previously reported protocol with some modifications (51). Briefly, the hESC culture medium was changed to mTeSR1 Plus containing 1× CloneR2 (STEMCELL Technologies), 2 hours before electroporation. The ribonucleoprotein (RNP) complex was prepared by mixing Alt-R SpCas9-GFP V3 (IDT), 1 pair of Alt-R sgRNAs targeting each ICR (IDT), Alt-R Cas9 Electroporation Enhancer (IDT), and P3 solution with supplement (Lonza). Following detachment using Accutase (STEMCELL Technologies), hESCs were spun down, resuspended with mTeSR1 Plus with 1× CloneR2, and mixed with the RNP complex. Electroporation was performed using the program CA-137 of a 4D Nucleofector (Lonza), and cells were maintained overnight in a CO_2_ incubator. On the following day, cells in the top 10% GFP-positive population were single-cell sorted into 96-well plates using a FACS AriaII cell sorter (BD Biosciences). Following the 10–14 days of culturing, every single clone was subcultured on 48-well plates, and wells with 2 or more colonies were excluded.

We also used HCT116 cells as an example of somatic cells because they had a near-normal karyotype (52). HCT116 cells were obtained from the American Type Culture Collection (ATCC) and maintained in McCoy’s 5A media (Gibco, Thermo Fisher Scientific) containing 10% FBS. HCT116 cells stably expressing Cas9 were generated by lentivirus-mediated transduction using lentiCas9-Blast (Addgene plasmid 52962), pMD2.G (Addgene plasmid 12259), and psPAX2 (Addgene plasmid 12260). To generate the STX16-ICR homozygously deleted HCT116 cells, we serially infected cells with lentiviruses encoding gRNAs and puromycin or hygromycin resistance cassettes, which were generated using lentiGuide-Puro (Addgene plasmid 52963) or lentiGuide-Hygro-EGFP (Addgene plasmid 99375), respectively. Infected cells were selected by each antibiotic, followed by single-cell sorting. NESP-ICR–deleted HCT116 cells were generated by gRNA delivery using Lipofectamine RNAiMax (Thermo Fisher Scientific) into cells stably expressing Cas9, followed by single-cell sorting.

### Genomic DNA preparation, PCR, Sanger sequencing, knockdown, and qRT-PCR.

Genomic DNA was extracted from hESCs or HCT116 cells using the DNeasy Blood and Tissue Kit (QIAGEN), and endpoint PCRs were performed using KOD One (TOYOBO). Sanger sequencing was performed with a cycle sequencing reaction using the BigDye, version 3.1, Cycle Sequencing Kit (Thermo Fisher Scientific) and analyzed with an ABI 3730XL DNA Analyzer (Thermo Fisher Scientific).

For knockdown experiments, siRNAs targeting OCT4 (Thermo Fisher Scientific, Silencer Select, s10872) and SOX2 (Thermo Fisher Scientific, Silencer Select, s13294) were used. Transfection of siRNAs was performed using TransIT X2 (Mirus Bio) following the manufacturer’s protocol. Seventy-two hours after transfection, cells were collected for RNA preparation.

RNA was extracted from hESCs or HCT116 cells using the RNeasy Kit (QIAGEN) and reverse transcribed using ProtoScript II Reverse Transcriptase (New England BioLabs). qRT-PCR was performed using KOD SYBR (TOYOBO) in a QuantStudio 3 real-time PCR system (Thermo Fisher Scientific). Relative expression levels were calculated by the ΔΔCt method using *ACTB* as an internal control gene. Primer sequences are listed in Supplemental Table 3.

### DNMT1 inhibition and MSRE-qPCR.

A selective DNMT1 inhibitor, GSK3484862 (MedChemExpress), was dissolved in dimethylsulfoxide and added to the culture media at a final concentration of 2 μM, based on methods described in previous reports (24, 53). Forty-eight hours later, GSK3484862 was removed from the media by replacing it with fresh culture media.

Methylation quantification by MSRE-qPCR was performed as we previously described (23). Briefly, 150 ng genomic DNA was digested with 10 units of methylation-sensitive HpaII (New England BioLabs) at 37°C for 2 hours. Relative amounts of digested samples and serially diluted undigested samples were measured by qPCR with KOD SYBR (TOYOBO) using the QuantStudio 3 Real-time PCR system (Thermo Fisher Scientific). In every sample, a melt curve with a single peak was confirmed. Methylation levels were calculated on the basis of Ct values for digested samples, and the calibration curves were generated from Ct values for serially diluted undigested samples. Primer sequences are listed in Supplemental Table 3.

### Bisulfite PCR and amplicon sequencing analysis.

Bisulfite PCR and amplicon sequencing were performed as we previously described (45). Genomic DNA was bisulfite converted using the EZ DNA Methylation-Gold Kit (ZYMO RESEARCH) following the manufacturer’s protocol, and each target region was amplified by a single-step PCR using KOD One. Primer sequences are listed in Supplemental Table 3. PCR products were purified using the QIAquick PCR Purification Kit (QIAGEN) and were subjected to NGS analysis at the Massachusetts General Hospital DNA Core. FASTQ files were aligned to reference sequences that reflect bisulfite-converted sequences except for the CpG dinucleotides. The alignment was performed (54), and the aligned results, including the methylation levels, were visualized using Integrative Genomics Viewer, version 2.3 (Broad Institute).

### Luciferase assay.

Backbone plasmids were gifts from Tatsuya Kobayashi (Massachusetts General Hospital). For vector construction, the NESP55 promoter region (GRCh37 chr20:57,413,458-57,415,075) was PCR amplified and subcloned into the NcoI site of the pGL4.10 firefly luciferase–encoding vector. The region including the STX16-ICR (GRCh37 chr20:57,244,087-57,245,984) or a negative control region (GRCh37 chr20:57,252,945-57,256,022) was PCR amplified and subcloned between SalI and BamHI sites of the NESP55 promoter–inserted pGL4.10 vector. We chose these cloning sites so that the STX16-ICR and NESP55 promoter sequences were separated by 2 kb, including a poly(A) signal and a transcriptional pause site, as previously described (55). Truncated constructs of the STX16-ICR were generated by inverse PCR followed by ligation. Each truncated construct contained the following chromosomal regions: TR1, GRCh37 chr20:57,244,524-57,245,984; TR2, GRCh37 chr20:57,244,729-57,245,984; TR3, GRCh37 chr20:57,244,087-57,245,119; and TR4, chr20:57,245,460-57,245,984. The OCT4/SOX2 binding site mutant or deleted vectors were generated by inverse PCR followed by ligation.

For luciferase assays, vectors were introduced into hESCs or HCT116 cells by lipofection using Lipofectamine 3000 (Thermo Fisher Scientific) following the manufacturer’s protocol. Cells were lysed 72 hours after transfection, and luminescence was measured using the Dual-Glo Luciferase Assay System (Promega) and ENVISION (PerkinElmer). Firefly luciferase counts were normalized to the *Renilla* luciferase counts.

### 3C assay and parental determination of interacting allele.

The 3C assay was performed following a previously reported protocol (56) with some modifications. Four to 5 million hESCs were crosslinked in 1% formaldehyde for 10 minutes at room temperature, followed by quenching with the addition of glycine at a final concentration of 125 mM. Cells were then lysed in lysis buffer (10 mM Tris-Cl, pH 8.0, 10 mM NaCl, 0.2% NP-40, and 1× protease inhibitor cocktail) for 15 minutes on ice. After homogenization, nuclear pellets were collected by centrifugation at 2,500*g* for 5 minutes at room temperature, and the pellets were washed with 1× rCutSmart buffer (New England BioLabs). Chromatins were denatured by incubation at 65°C for 10 minutes in the presence of SDS at a final concentration of 0.1%. Then, Triton X-100 (Thermo Fisher Scientific) at a 1% final concentration was added to sequester SDS, and chromatins were digested with HindIII-HF (New England BioLabs) at 37°C overnight. HindIII was inactivated by adding SDS at a final concentration of 1.6% and incubation at 65°C for 30 minutes. The proximity ligation of digested chromatins was then performed using 15-fold-diluted chromatins and 5,000 U/mL T4 ligase (New England BioLabs) at 16°C for 3 hours in 1× T4 ligase buffer, 1% Triton X-100, 0.1 mg/mL BSA, and 1 mM ATP. Chromatin was reverse crosslinked by proteinase K at 65°C overnight, and DNA was purified using the QIAquick PCR Purification Kit (QIAGEN). For 3C PCR, we designed all primer sets so that they were oriented in the same direction, as recommended in the literature (56). Primer sequences are listed in Supplemental Table 3.

The parental origin of the interacting allele was determined by amplicon sequencing of the 3C PCR products. The PCR products were purified using the QIAquick PCR Purification Kit and analyzed by NGS at the Massachusetts General Hospital DNA Core. FASTQ files were aligned to reference sequences using the BWA-MEM algorithm on the Galaxy Platform 1 (54). Maternal and paternal allelic frequency was calculated on the basis of A (maternal) and G (paternal) read counts at rs3787497, respectively.

### CUT&RUN qPCR.

CUT&RUN qPCR (57) was performed using the CUT&RUN Assay Kit (Cell Signaling Technology) following the manufacturer’s protocol. We used antibodies against OCT4 (Cell Signaling Technology, no. 2750), SOX2 (Cell Signaling Technology, no. 23064), and normal rabbit IgG (Cell Signaling Technology, no. 66362). Briefly, approximately 10^5^ hESCs were captured by 10 μL concanavalin A beads and permeabilized with digitonin. Anti-OCT4 and anti-SOX2 (2 μL) or anti–normal rabbit IgG (5 μL) antibodies were added to 200 μL cell suspension in antibody-binding buffer and incubated at 4°C overnight. After that, protein A/G micrococcal nuclease (MNase) was added to antibody-bound permeabilized cells. Following calcium-dependent activation of MNase, cells were incubated at 4°C for 30 minutes, which was stopped by adding the Stop Buffer. Genomic DNA fragments were eluted at 37°C for 10 minutes and purified using columns. Input samples were prepared from the cell suspension before the addition of antibodies, and DNA was purified using columns and after sonication.

Relative amounts of CUT&RUN samples were calculated by qPCR using KOD SYBR (TOYOBO) in the QuantStudio 3 Real-time PCR system (Thermo Fisher Scientific). Primers targeting the OCT4/SOX2 site in the STX16-ICR are listed in Supplemental Table 3. We used commercially available primers targeting the *NANOG* promoter (Cell Signaling Technology, 95064) and αSatellite (Cell Signaling Technology, 4486) as a positive and a negative control locus, respectively. In every sample, a single-peak melt curve was confirmed. Relative amounts of the antibody-bound genomic region were calculated on the basis of the Ct values of CUT&RUN samples and the calibration curves generated from the Ct values of the serially diluted input samples.

### Western blotting.

Whole-cell lysates were extracted from hESCs and HCT116 cells using RIPA buffer (Thermo Fisher Scientific) following the manufacturer’s protocol, and protein concentrations were measured using the bicinchoninic acid assay. Whole-cell lysates (7 μg) were run on SDS-PAGE. Proteins were transferred onto a polyvinylidene fluoride membrane, and after blocking, target proteins were detected using the anti-OCT4 and anti-SOX2 antibodies described above. Anti–β-tubulin antibody (Cell Signaling Technology, 5346) was used as a loading control.

### Statistics.

Statistical analyses were performed using GraphPad Prism 9 (GraphPad Software). Individual data points are presented along with the mean ± SEM. The sample size, number of biological replicates, number of independent experiments, and statistical method are indicated in the figure legends. Outlier data points were not excluded. A 1-sample *t* test was used to compare data from WT cells and a group of independent clones or to compare allele fraction data with the predicted biallelic (0.5) value, followed by a Bonferroni correction for multiple comparisons. One-way ANOVA with Tukey’s post hoc test was used to compare multiple groups. The χ^2^ test was used to compare total allelic read counts in bisulfite sequencing. A *P* value of less than 0.05 was defined as statistically significant. *P* values of 0.05 or higher were considered nonsignificant.

### Study approval.

All experiments were approved by the Institutional Biosafety Committee of Mass General Brigham (no. 2019B000050).

## Author contributions

YI and MB conceived the study, designed the experiments, and interpreted data. YI, CA, and QH conducted cellular experiments. YI and MR performed methylation analyses. YI and BA conducted sequencing and bioinformatics analyses. MB obtained research funding for the study and supervised the experiments. YI drafted the manuscript, and MB and YI edited the manuscript with input from all the authors.

## Supplementary Material

Supplemental data

## Version 1 02/28/2023

In-Press Preview

## Version 2 04/17/2023

Electronic publication
