# Supplementary material for: The long-range interaction between two GNAS imprinting control regions delineates pseudohypoparathyroidism type 1B pathogenesis
Source: J Clin Invest. 2023 Apr 17;133(8):e167953. doi: 10.1172/JCI167953 (PMC10104902; doi:10.1172/JCI167953)
Supplement: Supplemental data [file jci-133-167953-s188.pdf]

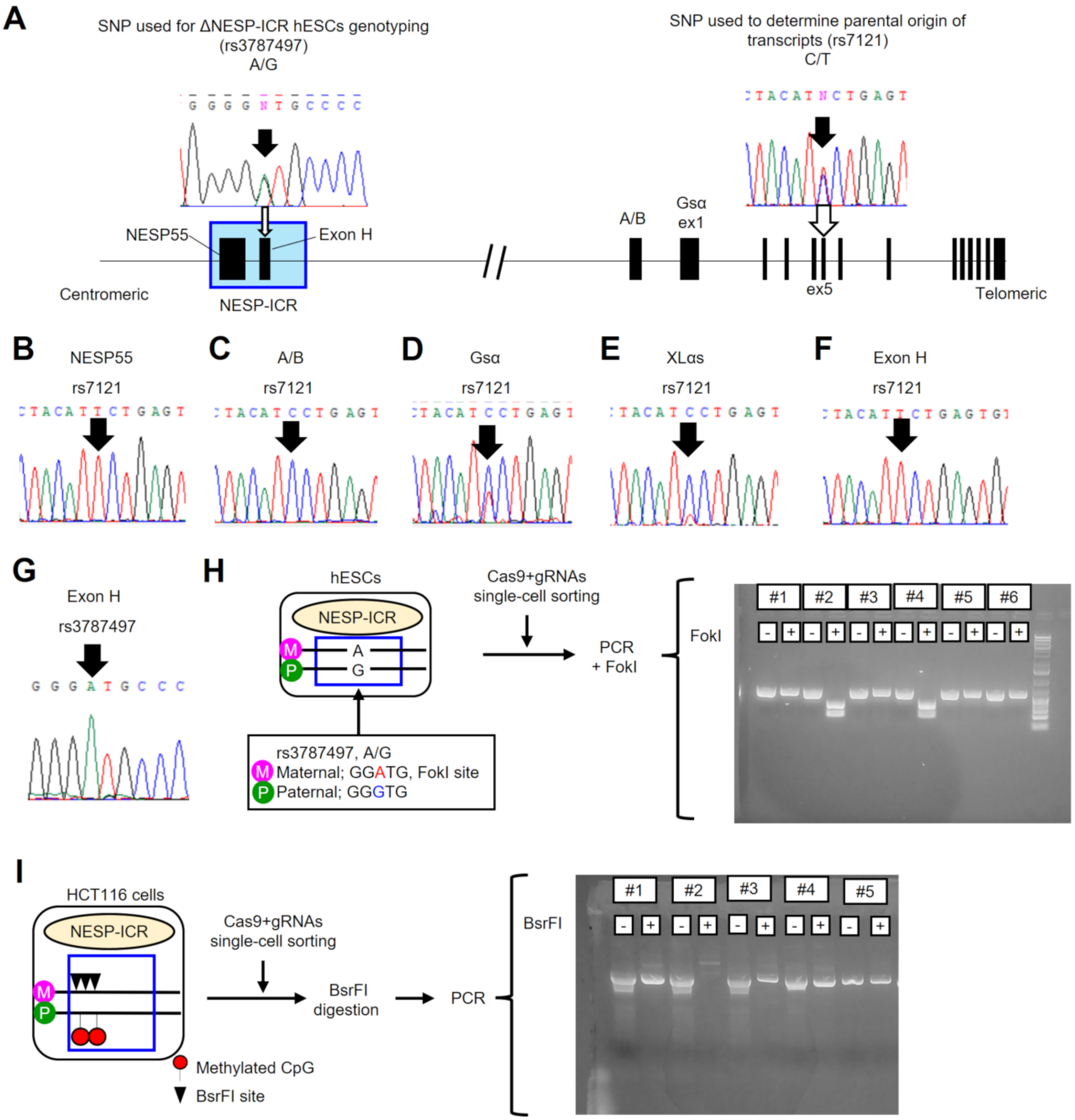

Supplemental Figure 1. Parental determination of the remaining allele in NESP-ICR deleted hESCs and HCT116 cells.

(A) SNPs utilized for allelic determination in NESP-ICR in hESCs. Genomic sequencing results of an SNP within exon H (rs3787497, left) and another SNP in *GNAS* exon 5 (rs7121, right) are shown.

(B-F) Sanger sequencing results of *GNAS*-derived transcripts. Each *GNAS*-derived transcript was amplified from hESC-derived cDNA using a specific forward primer in each exon and a common reverse primer in *GNAS* exon 7. (B-E) *GNAS* exon 5 SNP (rs7121) in NESP55 (B), A/B (C), Gsα (D), XLαs (E), and exon H (F). (G) Exon H SNP (rs3787497) in the exon H-containing transcript. (H) The genotyping workflow for NESP-ICR deleted hESCs. (Left) An SNP within exon H (rs3787497), which generates a FokI site only on the maternal allele, was used to determine parental alleles. (Right) PCR-restriction fragment length polymorphism for the parental determination of the remaining NESP-ICR allele. The exon H-surrounding region in NESP-ICR was PCR-amplified from genomic DNA, followed by FokI digestion. Digested PCR products indicate maternally remaining (paternally deleted) clones. A representative gel is shown.

(I) The genotyping workflow for NESP-ICR deleted HCT116 cells. (Left) The NESP55-surrounding region in NESP-ICR is paternally methylated. BsrFI, a CpG methylation-sensitive restriction enzyme, was used to digest the maternal allele. (Right) Following BsrFI digestion, the

NESP55-surrounding region was PCR-amplified. The amplification from the BsrFI-digested genome indicates paternally remaining (maternally deleted) clones. A representative gel is shown.

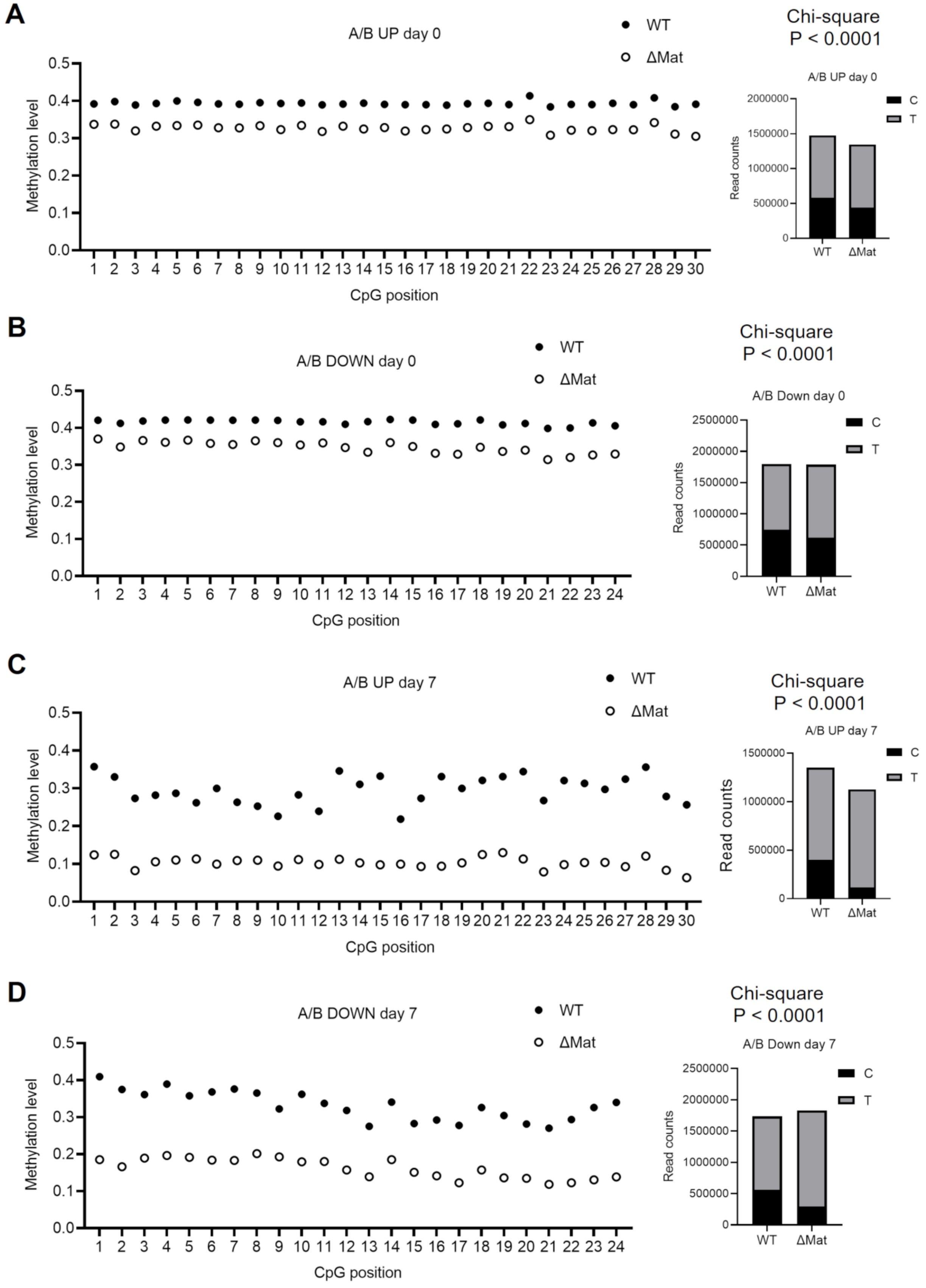
A
A/B UP day 0
WT
ΔMat
Methylation level
CpG position
Chi-square
P < 0.0001
A/B UP day 0
Read counts
C
T
WT
ΔMat
B
A/B DOWN day 0
WT
ΔMat
Methylation level
CpG position
Chi-square
P < 0.0001
A/B Down day 0
Read counts
C
T
WT
ΔMat
C
A/B UP day 7
WT
ΔMat
Methylation level
CpG position
Chi-square
P < 0.0001
A/B UP day 7
Read counts
C
T
WT
ΔMat
D
A/B DOWN day 7
WT
ΔMat
Methylation level
CpG position
Chi-square
P < 0.0001
A/B Down day 7
Read counts
C
T
WT
ΔMat

Supplemental Figure 2. Bisulfite sequencing analysis of A/B DMR in NESP-ICR deleted hESCs.

(A-D) In the experiment of Fig. 2D to G, genomic DNA was purified at days 0 and 7 from WT and a NESP-ICR ΔMat clone and was bisulfite-converted. The A/B DMR was PCR-amplified, and purified products were subjected to next-generation sequencing analysis. FASTQ data were aligned to reference sequences reflecting bisulfite-conversion except for the CpG dinucleotides. The methylation level at each CpG site is shown on the left panel. Total read counts of C (methylated) and T (unmethylated) are shown on the right panel. WT and ΔMat clones were compared using the chi-square test.

(A) A/B upstream (UP), day 0. (B) A/B downstream (DOWN), day 0. (C) A/B UP, day 7. (D) A/B DOWN, day 7.

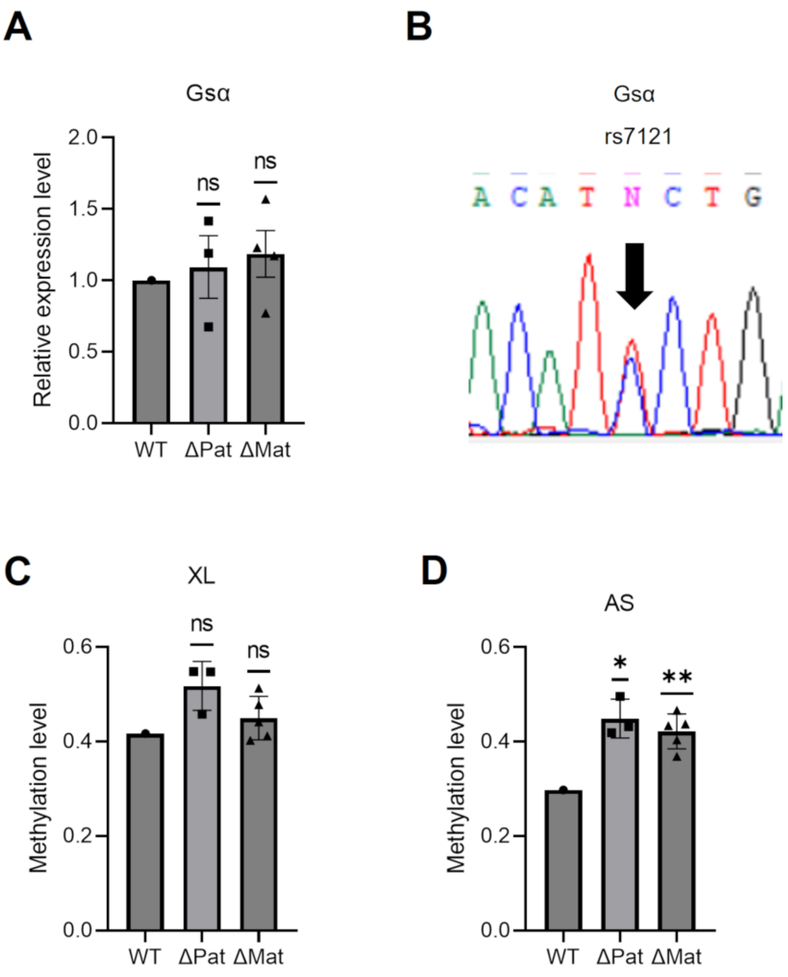

Supplemental Figure 3. Additional transcript and methylation analyses in NESP-ICR deleted hESCs.

(A) Expression levels of the Gsα transcript in WT, NESP-ICR paternally deleted (ΔPat, three independent clones), and maternally deleted (ΔMat, five independent clones) hESCs, quantified by qRT-PCR, normalized to β-actin.

(B) Sequencing of a *GNAS* exon 5 SNP (rs7121) in Gsα transcripts in NESP-ICRΔMat hESC clones. Four clones were analyzed, and a representative result is shown.

(C and D) Methylation analysis of WT, NESP-ICR paternally deleted (ΔPat, three independent clones), and maternally deleted (ΔMat, five independent clones) hESCs. Methylation levels at AS (C) and XL (D) DMRs were calculated by MSRE-qPCR.

For (A), (C), and (D), each dot represents the result of an independent hESC clone; WT vs. ΔMat or ΔPat clones were compared using a one-sample t-test followed by Bonferroni correction for multiple comparisons; * $p<0.05$, ** $p<0.01$, ns, not significant.

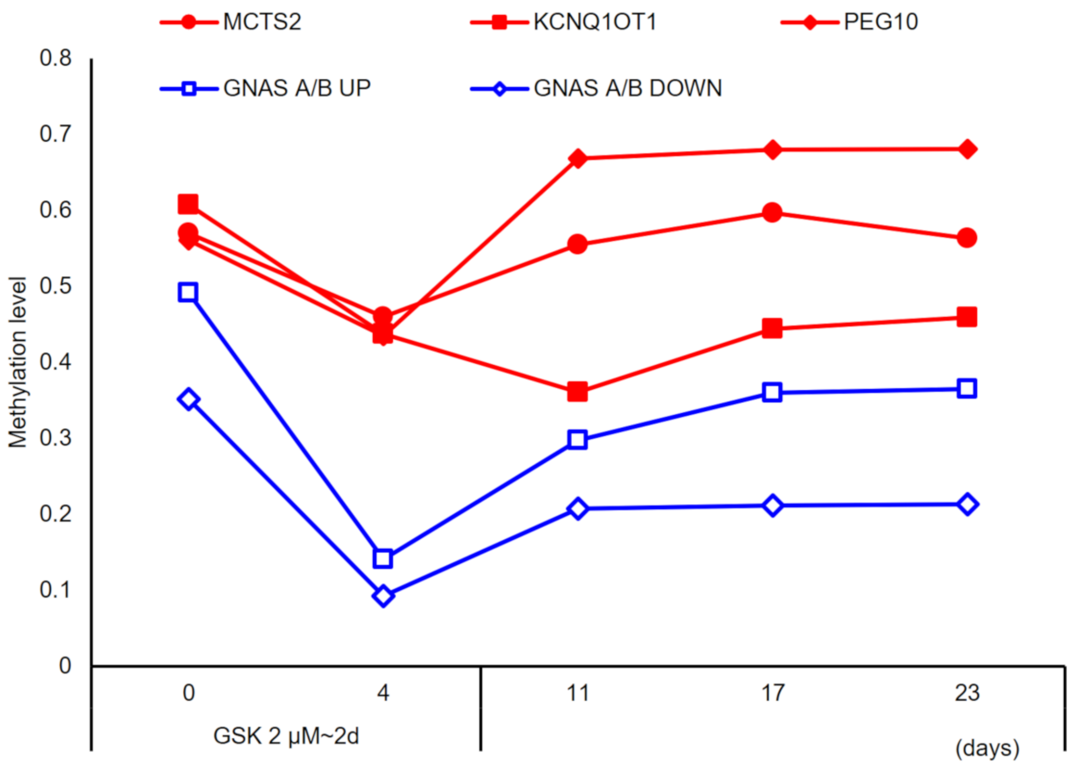

Supplemental Figure 4. GSK3484862-induced methylation changes at several imprinted loci in hESCs.

WT hESCs were treated with 2 μM GSK3484862 for 2 days. Following the removal of GSK3484862, genomic DNA was purified at the indicated time points, and methylation levels at upstream (UP) and downstream (DOWN) of A/B DMR, *MCTS2*, *PEG10*, and *KCNQ1OT1* were calculated by MSRE-qPCR.

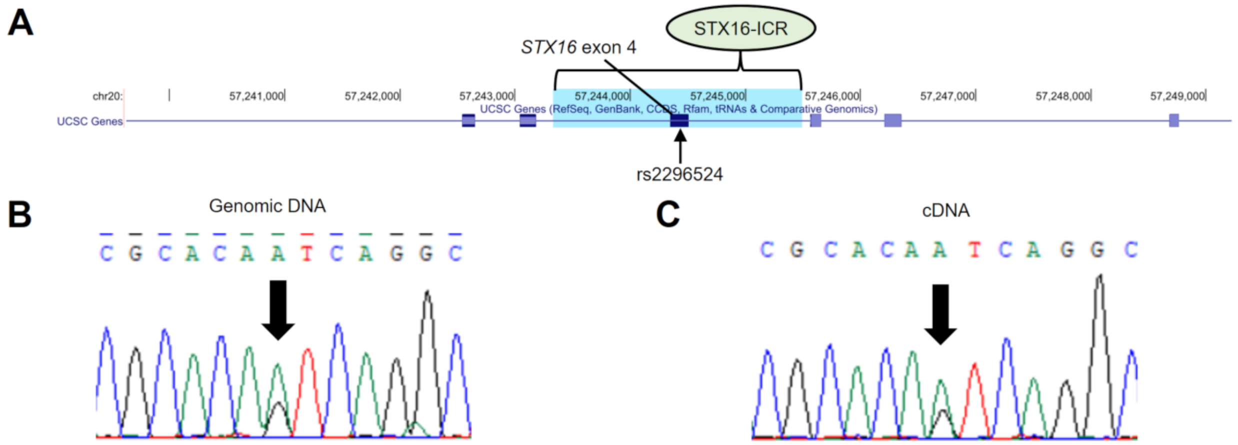

Supplemental Figure 5. Allelic origin of the *STX16* transcript in hESCs.

(A) Schematic location of the SNP (rs2296524) used for allelic determination. Exon numbering is based on the transcript NM_003763.6. STX16-ICR targeted by CRISPR/Cas9 is shown in blue highlight (GRCh37 chr20:57,243,339-57,245,500).

(B) Genomic DNA sequencing of rs2296524 in WT hESCs.

(C) Complementary DNA (cDNA) sequencing of rs2296524 in WT hESCs.

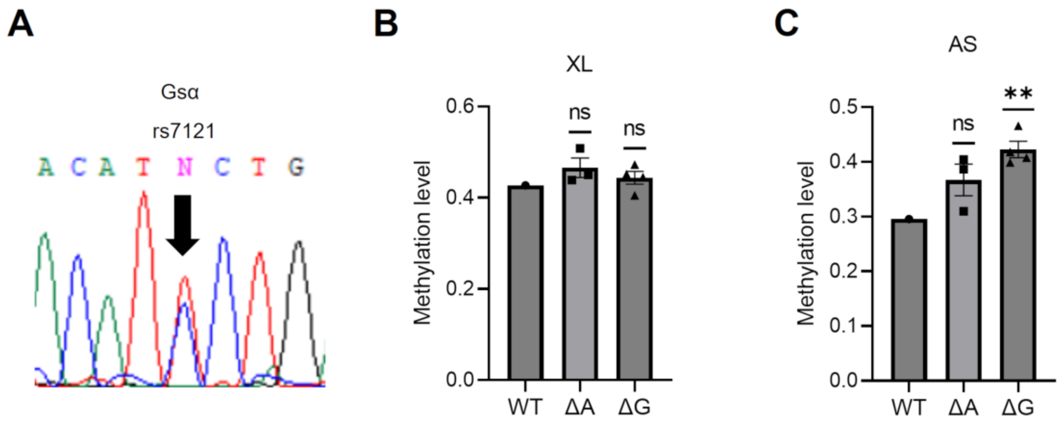

Supplemental Figure 6. Additional transcript and methylation analyses in STX16-ICR deleted hESCs.

(A) Sequencing of a *GNAS* exon 5 SNP (rs7121) in Gsα transcripts in STX16-ICRΔG clones. Three clones were analyzed, and a representative result is shown.

(B and C) Methylation analysis of WT, STX16-ICRΔA (three independent clones), and ΔG (four independent clones) hESCs. Methylation levels at AS (B) and XL (C) DMRs were calculated by MSRE-qPCR. Each dot represents the result of an independent hESC clone; WT vs. ΔA or ΔG clones were compared using a one-sample t-test followed by Bonferroni correction for multiple comparisons; ** $p<0.01$, ns, not significant.

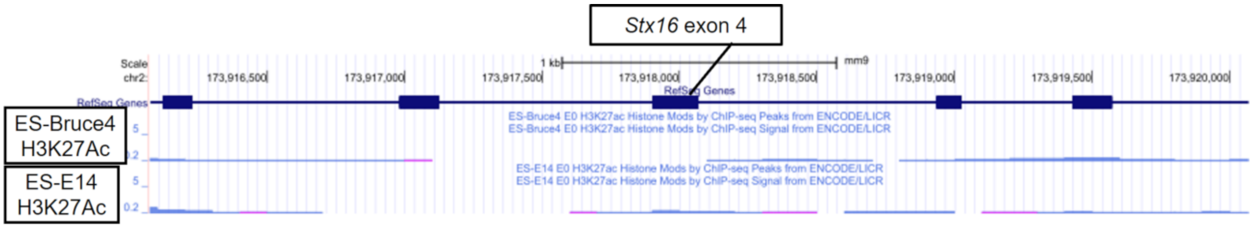

Supplemental Figure 7. Genome Browser tracks of mouse *Stx16* region.

H3K27Ac ChIP-seq signals in murine embryonic stem cells are shown (mm9). Exon numbering is based on the transcript NM_ 001102423.1.

A

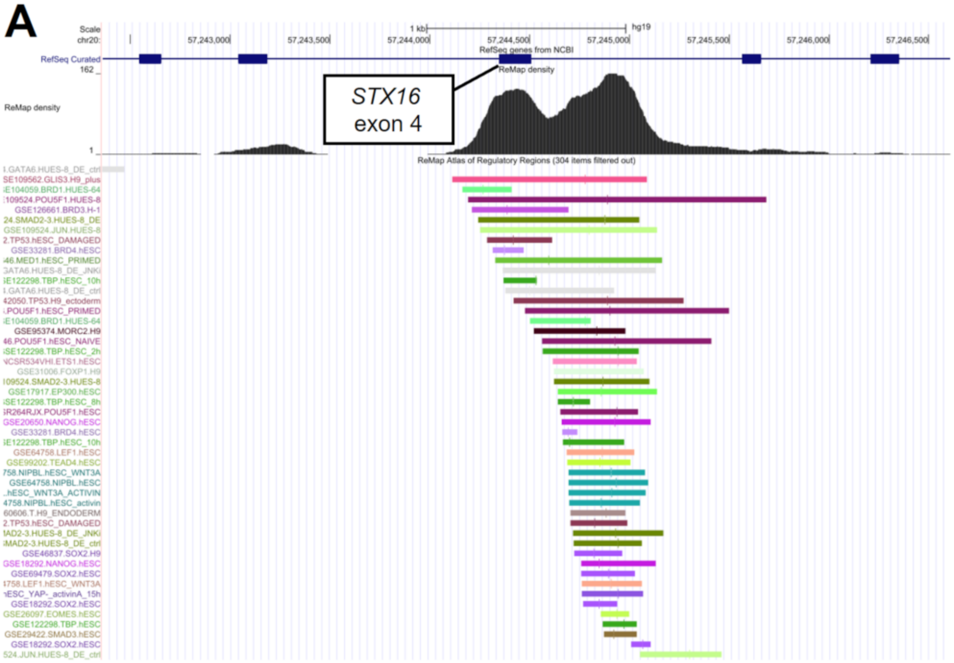

B

Factors with ChIP-seq signal in STX16-ICR
GLIS3, BRD1, OCT4, BRD3, SMAD2/3, JUN, TP53, BRD4, GATA6, MED1, TBP, MORC2, ETS1, FOXP1, EP300, NANOG, LEF1, TEAD4, NIPBL, TBXT, SOX2, EOMES

C

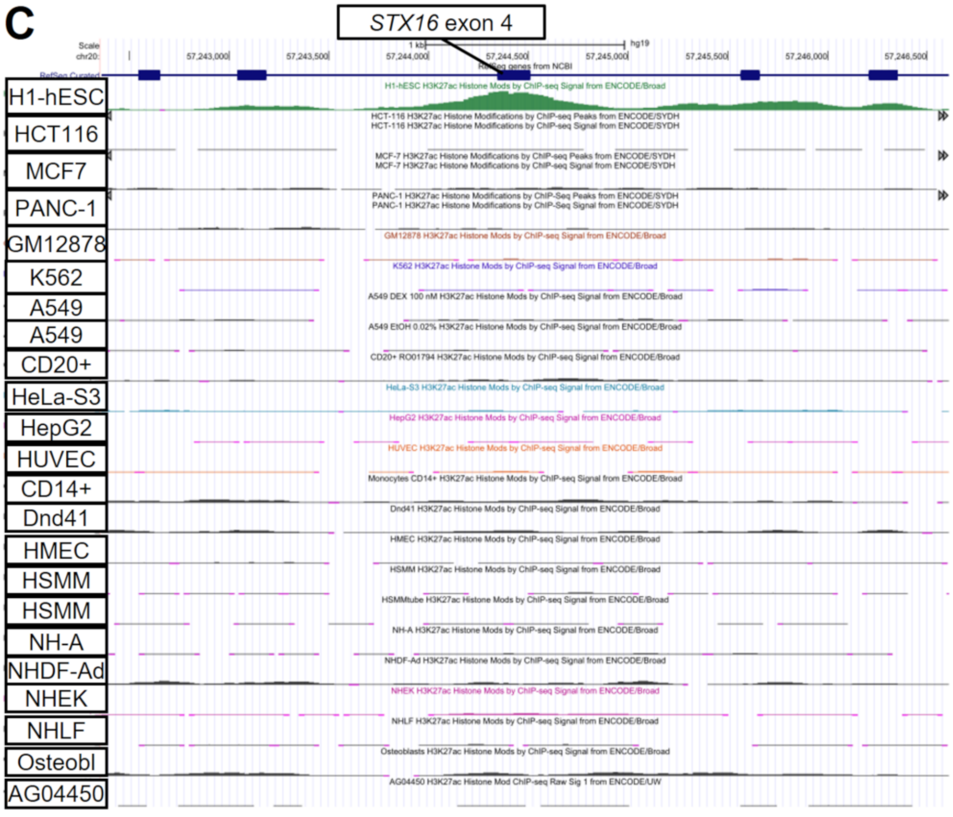

Supplemental Figure 8. Transcription factor recruitment and H3K27Ac mark in STX16-ICR of human cells.

(A and B) Transcription factor ChIP-seq signals. A genome browser track of hESCs (A) and the list of recruited transcription factors (B) are shown.

(C) A genome browser track showing H3K27Ac ChIP-seq signals surrounding STX16-ICR in hESCs and several human somatic cells.

For (A) and (C), exon numbering is based on the transcript NM_003763.6.

	Affected exons	Chromosomal Deletion (GRCh37 chr20)			*GNAS* methylation status				Notes	PMID	Ref
		Length	Centromeric breakpoint	Telomeric breakpoint	NESP	AS	XL	A/B			
①	NESP55, exon H, AS exon 3-4	4.0 kb	57,413,845	57,417,875	(Matdel)	L	L	L		15592469	20
②	NESP55, exon H, AS exon 3-4	4.7 kb	57,413,445	57,418,131	(Matdel)	L	L	L		15592469	20
③	NESP55, exon H	19.0 kb	57,397,711	57,416,700	(Matdel)	N	N	L		22378814	58
④	NESP55	9.5 kb	57,406,458-57,406,461	57,415,988-57,415,991	(Matdel)		N	L		34157100	18
⑤	NESP55, exon H, AS exon 3-5	37.6 kb + 1.4 kb	57,380,466<br>57,418,522	57,418,062<br>57,419,948	(Matdel)	L	L	L		26479409	59
⑥	Exon H, AS exon 3-4	4.2 kb	57,416,357	57,420,530	H	L	L	L		20444925	38
⑦	Exon H	40 bp	57,416,653	57,416,693	H	L	L	L	#	25005734	60
⑧	Intron 1 of NESP55/exon H, intron 2 of AS	33 bp	57,418,256	57,418,290	N	L	L	L	§	25005734	60

Supplemental Table 1. Detailed information of microdeletions reported in AD-PHP1B patients with NESP-ICR deletion (corresponding to Figure 1B).

Notes; #, Incomplete cosegregation of maternal deletion with *GNAS* methylation defects. §, Unknown inheritance, some AHO-like features were present.

	Affected exons	Chromosomal Deletion (GRCh37 chr20)			*GNAS* methylation status				PMID	Ref
		Length	Centromeric breakpoint	Telomeric breakpoint	NESP	AS	XL	A/B		
①	*STX16* exon 4-6	3.0 kb	57,243,567-57,243,739	57,246,545-57,246,717	N	N	N	L	14561710	19
②	*STX16* exon 2-4	4.4 kb	57,240,483-57,240,485	57,244,851-57,244,853	N	N	N	L	15800843	27
③	*STX16* exon 2-8	24.6 kb	57,235,162	57,259,753	N	N	N	L	24438374	61
④	Whole *STX16* and *NPEPL1*	87.5 kb deletion with 28 bp insertion	57,215,898	57,301,636	N	N	N	L	32337648	62
⑤	Whole *STX16* and *NPEPL1*	206 kb	57,151,892-57,289,110	57,289,120-57,358,140	N		N	L	34157100	18

Supplemental Table 2. Detailed information of microdeletions reported in AD-PHP1B patients with STX16-ICR deletion (corresponding to Figure 1C).

MSRE-qPCR	A/B upstream	Fw	GATTTTTCGCGCTTCCCCTTC
		Rv	GCCGACGCGACTGAGTG
	A/B downstream	Fw	TTTGGCGCTAACTCTTAGGCAGC
		Rv	CTTCATGGCCATCTTCAGCATGG
	AS	Fw	AGTGGGGCTAAAGGAGCTGAC
		Rv	TTGGGGTTTAATGCCGGTTTAG
	XL	Fw	CAGAGAGACCCCCAGTTGAG
		Rv	ATCGGCAGCCTGGATCTCG
qRT-PCR	hACTB	Fw	CACCCAGCACAATGAAGATC
		Rv	GTCATAGTCCGCCTAGAAGC
	NESP55	Fw	AAGAGTCGAAGGAGCCCAAGGAG
	Exon H	Fw	AAAGTACCTGGGGGAAAGGTAG
	XL	Fw	AGAAGCGCGCAGAGAAGAAACG
	A/B	Fw	CTTGCGTGTGAGTGCACCTC
	Gsα exon1	Fw	CAGAAGGACAAGCAGGTCTACC
	GNAS exon 2 (shared)	Rv	CCATTAAACCCATTAACATGCAG
	SOX2	Fw	CTGCAGTACAACTCCATGACCAG
		Rv	TGCGAGTAGGACATGCTGTAGG
	OCT4	Fw	TTCAAGAACATGTGTAAGCTGCG
		Rv	ACTCGGTTCTCGATACTGGTTC
Bisulfite PCR	A/B upstream	Fw	AAAATTGGGAGGTAGGTTTGGGAG
		Rv	CCCCAACCTCTTCAAAAAACC
	A/B downstream	Fw	TTAATTTTTAGGTAGTTAGTTTAGTAGTT
		Rv	TAAACTTCATAACCATCTTCAACATAA
3C-PCR	primer #1		TTAGACTTGGGTCCCATCCAGAATATCTC
	primer #2		GGACCTGGTATTCCCTGACAAACATTGC
	primer #3		TGTGCGGAAAGTAATCTGAATGGG
CUT&RUN qPCR	STX16 intron 4 OCT4/SOX2	Fw	GAGCTGCTCTTCAATAGGTAAAAAGC
		Rv	GGTTGTTATGCAAATATGTGGCTTTCAG

Supplemental Table 3. Primer sequences.

References for supplementary materials

58. Richard N et al. A new deletion ablating NESP55 causes loss of maternal imprint of A/B *GNAS* and autosomal dominant pseudohypoparathyroidism type Ib. *J Clin Endocrinol Metab*. 2012;97(5):E863-867.

59. Takatani R et al. Analysis of Multiple Families With Single Individuals Affected by Pseudohypoparathyroidism Type Ib (PHP1B) Reveals Only One Novel Maternally Inherited *GNAS* Deletion. *J Bone Miner Res*. 2016;31(4):796–805.

60. Rezwan FI et al. Very small deletions within the NESP55 gene in pseudohypoparathyroidism type 1b. *Eur J Hum Genet*. 2015;23(4):494–499.

61. Elli FM et al. Autosomal dominant pseudohypoparathyroidism type Ib: a novel inherited deletion ablating STX16 causes loss of imprinting at the A/B DMR. *J Clin Endocrinol Metab*. 2014;99(4):E724-728.

62. Yang Y et al. A novel long-range deletion spanning *STX16* and *NPEPL1* causing imprinting defects of the *GNAS* locus discovered in a patient with autosomal-dominant pseudohypoparathyroidism type 1B. *Endocrine*. 2020;69(1):212–219.
